# Prenatal exposure to asthma medications and risk of neurodevelopmental disorders and educational difficulties: A systematic review and meta-analysis

**DOI:** 10.1371/journal.pmed.1005100

**Published:** 2026-06-01

**Authors:** Lama A. Shakhshir, Alexia Karain, Jill P. Pell, Claire E. Hastie, Scott M. Nelson, Michael Fleming

**Affiliations:** 1 School of Health and Wellbeing, University of Glasgow, Glasgow, United Kingdom; 2 School of Medicine, Dentistry and Nursing, University of Glasgow, Glasgow, United Kingdom; University of Cambridge School of Clinical Medicine, UNITED KINGDOM OF GREAT BRITAIN AND NORTHERN IRELAND

## Abstract

**Background:**

Since asthma exacerbations during pregnancy risk maternal and fetal health, continued medication is important. However, some studies have reported adverse neurodevelopmental outcomes following prenatal exposure to asthma medication. Therefore, this systematic review aimed to collate the existing evidence on the associations between prenatal exposure to asthma medication and neurodevelopmental and educational outcomes.

**Methods and findings:**

A systematic review was conducted in accordance with PRISMA guidelines and the PECO framework. PubMed, Medline and Embase databases were searched for studies investigating prenatal exposure to one or more asthma medication and neurodevelopmental or educational outcomes published, in English, between January 2003 and September 2024, and updated in November 2025. Studies of asthma medication used for other indications were excluded. Study quality was assessed using the Newcastle-Ottawa scale. Random-effects meta-analyses were conducted where appropriate and heterogeneity was evaluated using Cochran’s *Q* and *I*^2^ tests.

Of 16,824 studies identified by the initial search, seven were eligible for inclusion. All investigated beta-2-adrenergic agonists (B2AA), with one including B2AA as mono- and polytherapy—and one study also investigated inhaled corticosteroids (ICS) exposure. Two reported associations with autism spectrum disorder (ASD) and one with attention-deficit hyperactivity disorder (ADHD). An updated search identified one additional eligible study, which examined both ADHD and ASD, as well as other neurodevelopmental disorders. The included eight studies (*n* = 3,867,170 participants) comprised cohort (*n* = 5) and case-control (*n* = 3) designs and reported inconsistent results. Meta-analysis of three studies (*n* = 1,380,871) indicated significant associations with ASD for exposure to B2AA both preconception (aOR 1.34, 95% CI [1.19,1.52]) and during pregnancy (aOR 1.29, 95% CI [1.16,1.42]). Heterogeneity was low, with no evidence of significant publication bias. Limitations of the included studies comprised residual confounding and exposure misclassification. Additionally, studies included in the meta-analysis were few in number and did not adequately distinguish between medication effects and underlying maternal asthma.

**Conclusion:**

Meta-analysis suggested an association between prenatal exposure to B2AA and ASD. An association with ADHD, reported in a single study, requires corroboration. To date, based on our search strategy, no association has been reported with communication skills, motor skills, problem-solving and personal-social skills, or cerebral palsy.

## Author summary

**Why was the study done?:**

- Asthma is a common chronic condition characterized by exacerbations during pregnancy which, if untreated, could lead to harmful effects on both the mother and her child.
- Several studies in the literature highlighted the safety of asthma medication when it comes to early pregnancy outcomes. However, limited knowledge is available regarding the subsequent neurodevelopmental and educational outcomes the children might experience following prenatal exposure to these medications.
- The evidence generated from this systematic review can inform policy decisions and support broader impacts on practice and outcomes.

**What did the researchers do and find?:**

- Conflicting results were found when papers investigating these association were collated.
- Meta-analysis was conducted on the studies that investigated the same outcome and exposure. Prenatal exposure to beta-2-adrenergic agonists (B2AA) was found to be associated with a higher risk of autism spectrum disorder (ASD).

**What do these findings mean?:**

- Prenatal exposure to beta-2 agonists may be associated with a higher risk of ASD, but further studies are needed to confirm whether this reflects a treatment effect or confounding by indication or severity.
- Women with asthma require ongoing management during pregnancy, but treatment decisions need to be informed by evidence of the benefits and risks of different treatment options.
- The generalizability of the findings regarding the meta-analysis is limited by the few numbers of studies included (*n* = 3) and their insufficient differentiation between the disease and medication effects.

## Abstract 3

In a systematic review, Lama A. Shakhshir and colleagues assess the available evidence on prenatal exposure to asthma medications and subsequent neurodevelopmental and educational outcomes in children.

## Introduction

Asthma affects 8%–13% of pregnant women globally, making it one of the most common chronic conditions encountered during pregnancy. Since exacerbations occur more frequently during pregnancy [1] and can be harmful for both the mother and the fetus [2], the Global Initiative for Asthma (GINA) guidelines explicitly recommend continuing asthma medication throughout pregnancy [3]. GINA proposes two asthma treatment tracks, each organized into five steps. Track 1 uses a combined inhaled corticosteroid (ICS)-formoterol inhaler for symptoms relief in the early steps, and then for both daily control and relief in later steps, with escalation to stronger doses or add-on therapies at Step 5 if control is not achieved. Track 2 is recommended when ICS-formoterol is not appropriate. It relies on short-acting beta-2-adrenergic agonist (SABA) for quick relief at all steps, with an ICS added at Step 1 and daily controller medicines increased progressively, from low-dose ICS to ICS-long-acting beta-2-adrenergic agonist (ICS-LABA) combinations, and then to higher-dose regimens with potential add-on therapies at the final step.

During pregnancy, asthma medications can cross the placenta, and their pharmacokinetics and pharmacodynamics undergo significant changes [4]. There is a reduction in the concentration of plasma proteins that bind to the drug making it inactive [5]. For example, albumin, the most abundant plasma protein, decreases 1% by 8 weeks of pregnancy, 10% by 20 weeks and 13% by 32 weeks [5]. Due to this reduced binding, the amount of free, active drug is increased in the maternal circulation and, consequently, in the body tissues. Therefore, asthma control and lung function should be assessed monthly in pregnant women with therapy modified as required [6].

Several studies have examined associations between prenatal exposure to asthma medication and pregnancy outcomes. Eltonsy colleagues’ systematic review of beta-2-adrenergic agonists (B2AA) exposure found that birthweight centile was lower among infants exposed to salmeterol (a LABA) compared to those exposed to budesonide (an ICS) [7]. However, no associations were observed between B2AA exposure and either preterm delivery or gestational age at delivery [7]. One study reported that first-trimester exposure to SABAs was associated with higher risk of cleft palate and gastroschisis [8].

There is a paucity of evidence regarding prenatal exposure to ICS and long-term neurodevelopment in children. Most existing studies focused instead on systemic antenatal corticosteroids (ACS) which are administered during preterm labor, to promote fetal lung maturation. A recent systematic review by Liauw and colleagues reported no associations between ACS exposure and neurodevelopmental outcomes across intellectual, motor, language, and social domains [9], but noted that some studies had observed modest reductions in non-verbal intelligence and visual memory [9]. In contrast, studies examining ICS specifically have focused primarily on early pregnancy outcomes. Systematic reviews by Lim and colleagues and Rahimi and colleagues found no association between ICS use and congenital anomalies, preterm labor, or low birth weight [10,11]. Similarly, Blais and colleagues reported no increased risk of congenital malformations following first-trimester exposure to ICS [12].

Many studies on longer-term outcomes have focused on maternal asthma rather than its treatment. Whilst some individual studies have reported an association between maternal asthma and autism spectrum disorder (ASD) in the offspring [13–15], a meta-analysis of 10 studies, that included 175,906 participants, reported no significant association [16]. Cortese and colleagues observed a significant association between maternal asthma and attention-deficit hyperactivity disorder (ADHD) in the offspring, after adjusting for potential confounders [17].

Studies specifically examining prenatal exposure to asthma medication have produced conflicting results. Some have reported an association between prenatal exposure to B2AA and neurodevelopmental disorders such as ASD and ADHD [18–20], while others found no significant associations [21–24]. The only previous systematic review on this topic focused exclusively on montelukast, a leukotriene antagonist (LTRA), and reported no increased risk of ADHD, ASD, or Tourette Syndrome [25].

Given the conflicting evidence and the lack of a comprehensive review of this important clinical question, this systematic review aims to collate and assess the available evidence on prenatal exposure to asthma medications and subsequent neurodevelopmental and educational outcomes in children.

## Methods

### Search strategy and inclusion criteria

A systematic literature review was undertaken using the PubMed, Medline (via Ovid), and Embase (via Ovid) databases. For each database, a search strategy was created using the Population Exposure Comparator Outcome (PECO) framework (Table A in S1 Appendix). The populations of interest were women, of no specified age, who took asthma medication during pregnancy, to manage the symptoms of asthma, and infants (1 day–1 year old), toddlers (1–3 years old), preschool children (3–5 years old), children (5–12 years old) and adolescents (13–18 years old) who had prenatal exposure to asthma medication. The exposure was defined as at least one type of asthma medication taken at any point during pregnancy, as a monotherapy or a part of a combination therapy. We selected, for inclusion, asthma medications that could be used during pregnancy according to the National Institute for Health and Care Excellence (NICE) guidelines. These included SABA, LABA, LTRA, ICS, oral corticosteroids (OCS), theophylline, and mast cell stabilizers (MCS).

The comparison group comprised the offspring of women who did not take any asthma medication during pregnancy. Two categories of outcomes were investigated: neurodevelopmental disorders and educational outcomes. Neurodevelopmental disorders were identified based on the 11th Revision of the International Classification of Diseases (ICD-11) for Mortality and Morbidity Statistics, developed by the World Health Organization (WHO), as well as relevant literature sources. We included conditions that occur during early childhood. These encompassed disorders of intellectual development, developmental speech or language disorder, ASD, developmental learning disorder, developmental motor coordination disorder, ADHD, and stereotypic movement disorder. Additionally, we included cerebral palsy because of its association with neurodevelopmental disorders and, therefore, its inclusion in many studies of neurodevelopmental outcomes [26–32]. Educational outcomes included academic attainment, school performance, and special educational needs (SEN) in children. The inclusion criteria comprised peer-reviewed papers, written in English, and published between 1 January 2003 and 1 September 2024, inclusive, to capture evidence relevant to contemporary clinical practice. The search was updated on 26 November 2025. The search was limited to English-language studies to minimize any potential translation-related inaccuracies. Studies were excluded if they investigated only maternal asthma and not asthma medications. Studies were also excluded if their results included women taking asthma medications for other indications; identified by explicit mention of B2AA use as a tocolytic agent to delay premature labor and/or mention of systemic administration of B2AA as an intravenous injection. Where results were stratified by indication or route of administration, results for the eligible sub-group were included. Studies with unclear inclusion/exclusion criteria were not included. Book chapters, editorials, commentaries, conference abstracts and review articles were excluded as well. The protocol of this review is registered on PROSPERO (CRD420251019228) and available from https://www.crd.york.ac.uk.

### Screening and data extraction

This study is reported as per the Preferred Reporting Items for Systematic reviews and Meta-Analyses (PRISMA) guidelines (Checklist A in S1 Appendix). Screening of titles, then abstracts, then full manuscripts were conducted. Two reviewers (LAS and AK) reviewed the articles independently of each other and, where decisions differed, consensus was reached via discussion. In cases where consensus could not be reached, a third reviewer was consulted to make the final decision. EndNote and Rayyan software were used to remove duplicate studies and support the screening process.

A standardized data extraction form was developed and piloted before full implementation. The data extracted from eligible studies and systematically tabulated included: year of publication, country, study design, sample size, sampling frame, recruitment method, inclusion/exclusion criteria, participant characteristics (maternal age, socioeconomic status, comorbidities), definitions and measurements of exposures (medication type, dosage, timing, and duration during pregnancy), outcome measures (assessment tools, diagnostic criteria, age at assessment), adjusted and unadjusted effect estimates with confidence intervals, and covariates included in analyses.

### Meta-analysis

Studies that measured the same exposure and outcome were meta-analyzed using random-effects models with restricted maximum likelihood (REML) to account for both within-study and between-study variance. REML was chosen as it functions well in most scenarios and produces unbiased non-negative estimates of Tau Squared (*τ*^2^) which indicates the magnitude of variability or heterogeneity between studies [33]. Five separate meta-analyses were conducted according to the stage of exposure: preconception, any time during pregnancy, 1st trimester, 2nd trimester, and 3rd trimester. Prior to pooling, all extracted effect estimates were examined for clinical and methodological heterogeneity by assessing differences in population characteristics, exposure definitions, outcome measurements, and statistical approaches. Statistical heterogeneity was assessed using Cochran’s *Q* test and quantified using the *I*^2^ measure, with <25%, 25%–50%, and >50% classified as low, moderate, and high heterogeneity, respectively [34]. For all analyses, adjusted effect estimates (odds ratios or incidence rate ratios) and their 95% confidence intervals (CIs) from individual studies were used to calculate pooled effect estimates and corresponding 95% CIs. Because included studies reported different measures of association (odds ratios and incidence rate ratios), we considered their comparability before pooling. Outcomes with a probability of less than 10% are considered rare [35]. Under the rare disease assumption, odds ratios can be interpreted as approximations of relative risks [35–37]. Relative risks and incidence rate ratios, while not identical, are closely related measures of association and are expected to be numerically similar under conditions of low incidence. Given that ASD had a global prevalence of 1% in 2021 [38], it was considered a rare outcome in our study. Hence, odds ratios and incidence rate ratios were considered sufficiently comparable and were combined in the meta-analyses. Potential publication bias was assessed using visual inspection of funnel plots and Egger’s regression test.

To test the robustness of our findings, we conducted sensitivity analyses by: removing the study with the smallest sample size, to check the consistency of results, and using fixed-effect models as an alternative analytical approach. All meta-analyses were conducted using Stata SE/18.0 software with the “meta” package.

### Quality assessment

The Newcastle-Ottawa Score was used to assess the methodological quality of the included studies. This validated tool evaluates different aspects of study design according to study type. For cohort studies, the tool covers three domains: selection (representativeness of the exposed cohort, selection of the non-exposed cohort, ascertainment of exposure, and demonstration that the outcome was not present at the start), comparability (of exposed and unexposed groups based on design and analysis), and outcome (assessment of outcome, adequacy of follow-up duration, and completeness of follow-up). For case-control studies, the domains include: selection (adequacy of case definition, representativeness of cases, selection of controls, and definition of controls), comparability (of cases and controls based on design and analysis), and exposure (ascertainment of exposure, same method of ascertainment for cases and controls, and non-response rate).

The scale uses a star system for quality assessment, with a maximum of 4 stars for the selection domain, 2 stars for the comparability domain, and 3 stars for the outcome/exposure domain. Studies were categorized as “good quality” if they scored 3 or 4 stars for selection, and 1 or 2 stars for comparability and 2 or 3 stars for outcome/exposure, “fair quality” if they scored 2 stars, 1 or 2 stars, and 2 or 3 stars respectively, and “poor quality” if they scored 0 or 1 star for selection or 0 stars for comparability or 0 or 1 star for outcome/exposure.

## Results

### Search results and study selection

**Fig 1:**
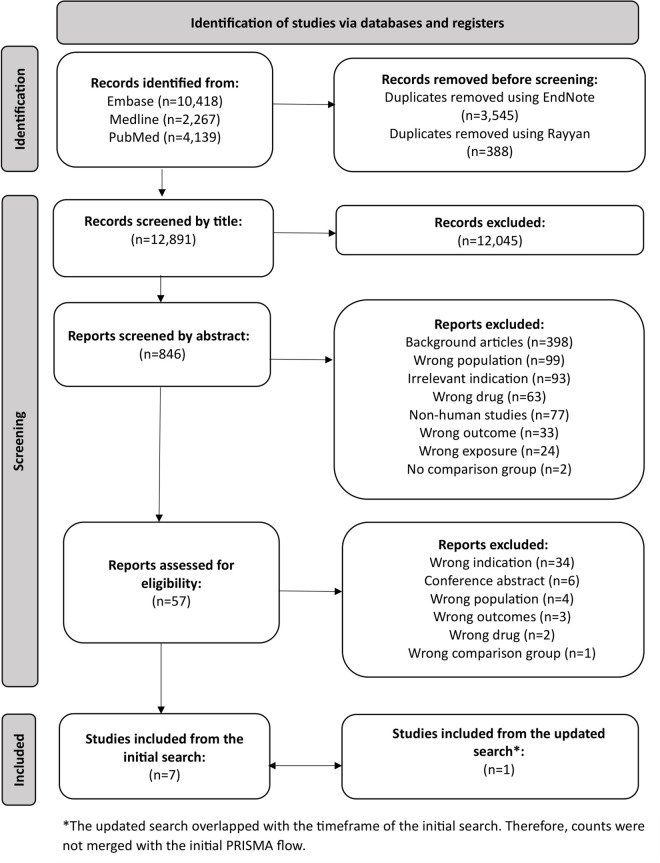
PRISMA chart.

The initial systematic search of PubMed, Medline, and Embase databases identified 16,824 articles published between January 1, 2003, and September 1, 2024. After removing 3,933 duplicates, 12,891 articles underwent title screening. Of these, 846 articles were selected for abstract review, and 57 for full-text assessment. Through manual searching of reference lists, an additional 310 potentially relevant articles were identified, though none met the inclusion criteria after screening. An updated search covering the period from January 1, 2024 to November 26, 2025 retrieved 1,880 records. Following removal of 469 duplicates, 1,417 records were screened, and one additional study met the inclusion criteria. Ultimately, eight studies were determined eligible for inclusion in this systematic review (Fig 1).

### Characteristics of included studies

The eight studies were published between 2011 and 2024, and all were conducted in high-income countries: four in Denmark (50%), and one each in the USA, Japan, Sweden, and Finland (Table 1). The total combined sample size across all studies was 3,867,170 participants. All studies employed observational designs; five (63%) were cohort studies and three (37%) were case-control studies (Table 1). Using the Newcastle-Ottawa scale for quality assessment, all five cohort studies and one case-control study were scored as good quality, representing 75% of the included studies (Table B in S1 Appendix). The remaining two case-control studies (25% of studies) were scored as fair quality. None were categorized as poor quality. Seven of the studies utilized routine administrative data sources such as national registries, and one study using data from an existing birth cohort (the Japan Environment and Children’s Study). All studies restricted their analyses to singleton births.

**Table 1:** Characteristics of the included studies.

Authors and years	Publication year	Study design	Location	Data source	Study population	*N* total	*N* cases	*N* exposed
Croen L. and colleagues [22]	2011	Case-control	California, USA	KPNC (Kaiser Permanente of Northern California) clinical databases	Singletons; 1 child per mother	575	291 ASD	575 beta-2 agonists
Gidaya N. and colleagues [20]	2016	Case-control	Denmark	Administrative data: Civil registration system	Singletons; 1 pregnancy per mother	628,408	5,200 ASD	1,679 beta-2 agonists
Su X. and colleagues [19]	2017	Cohort	Denmark	Administrative data: Civil registration system	Live singletons	751,888	9,098 ASD	29,211 beta-2 agonists
Gong T. and colleagues [21]	2018	Nested case-control	Sweden	Administrative data: Medical Birth Register	Live singletons	8,857	380 ASD	916 beta-2 agonists with or without other asthma medications
Liang H. and colleagues [18]	2017	Cohort	Denmark	Administrative data: Civil Registration System	Live singletons	672,265	573 ADHD	25,434 beta-2 agonists
Nagata A. and colleagues [24]	2023	Cohort	Japan	Birth cohort: Japan Environment and Children Study	Singletons, term deliveries	91,460	11,347 communication skills; 26,424 gross motor skills; 22,730 fine motor skills; 27,857 problem-solving; 11,775 personal-social	2,322 corticosteroids and/or beta-2 agonists
Li L. and colleagues [23]	2018	Cohort	Denmark	Administrative data: Danish Medical Birth Register	Live singletons	442,278	843 cerebral palsy	19,616 beta-2 agonists
Kemppainen M. and colleagues [39]	2024	Cohort	Finland	Administrative data: Finnish Medical Birth Register, the Drug Reimbursement Register, the Care Register for Health Care and the Register of Congenital Malformations	Live singletons	1,271,439	4,114 ADHD 1,617 ASD 1.569 motor development disorder 849 learning disabilities 3,057 language-developmental disorder 1,633 mixed developmental disorder 908 intellectual disability	88,626 asthma medication

N, number; ASD, autism spectrum disorder; ADHD, attention-deficit hyperactivity disorder.

### Exposures and outcomes in included studies

The studies investigated various neurodevelopmental outcomes, but none examined educational outcomes. Four studies investigated ASD, one ADHD, one cerebral palsy, and one failure to attain neurodevelopmental milestones. The latter included communication and motor problems, in addition to impaired personal and social skills. One investigated both ADHD and ASD, in addition to other neurodevelopmental disorders which comprised motor development disorder, learning disabilities, language-developmental disorder, mixed developmental disorder, and intellectual disability.

Five of the studies investigated prenatal exposure to B2AA as the primary medication of interest, while one study examined exposure to B2AA and/or corticosteroids [24], another assessed exposure to B2AA with or without exposure to other asthma medication [21], and one study examined the exposure to asthma medication more broadly [39]. Seven studies defined the exposure using the Anatomical Therapeutic Chemical (ATC) classification codes while one study relied on pharmacy databases (Table C in S1 Appendix). All eight studies investigated exposure at any time during pregnancy, with four additionally investigating exposure during each of the three trimesters separately to evaluate potential time-specific effects. Five studies examined preconception exposure, though they applied different definitions of the preconception period. These included 30 days prior to the last menstrual period [22], and 90 days [20] and one year prior to conception [19], as well as between two years and 30 days prior to the ‘beginning’ of the pregnancy [18,23]. Most studies relied on prescription records or claims to ascertain medication exposure. None of the studies had information on medication adherence or specific dosages used by pregnant women.

Among the four studies examining the association between ASD and B2AA exposure, two (50%) reported statistically significant associations at any time during pregnancy. One reported an odds ratio of 1.3 [95% CI 1.1,1.5] [20] whilst another reported an incidence rate ratio of 1.28 [95% CI 1.11,1.47] [19]. The other two studies found no associations [21,22]. The single study investigating ADHD [18] found a significant association with prenatal B2AA exposure at any time during pregnancy (Table 2). The study that investigated exposure to asthma medication and both ASD and ADHD reported an association with these disorders with hazard ratios of 1.42 [95% CI 1.28,1.58] and 1.54 [95% CI 1.44,1.65], respectively [39] (Table 2).

**Table 2:** Association between exposure to asthma medication and subsequent neurodevelopmental outcomes, by window of exposure.

Authors and years	Outcome	Preconception	Pregnancy as a whole	1st Trimester	2nd Trimester	3rd Trimester	Maternal covariates	Pregnancy covariates	Child covariates
Croen L. and colleagues (2011) [22]	ASD	OR 2.0 (95% CI [0.6,6.4])^a^	OR 1.2 (95% CI [0.7,2.0])^a^	OR 1.6 (95% CI [0.6,4.0])^a^	OR 1.4 (95% CI [0.6,3.1])^a^	OR 1.1 (95% CI [0.6,1.9])^a^	age; education	birth year; parity; gestational age; hospital	sex
Gidaya N. and colleagues (2016) [20]	ASD	OR 1.3 (95% CI [1.0,1.6])^b^	OR 1.3 (95% CI [1.1,1.5])^b^	OR 1.1 (95% CI [0.9,1.4])^b^	OR 1.5 (95% CI [1.1,1.7])^b^	OR 1.4 (95% CI [1.1,1.7])^b^	age; asthma	birth year and month	sex
Su X. and colleagues (2017) [19]	ASD	IRR 1.35 (95% CI [1.17,1.56])^c^	IRR 1.28 (95% CI [1.11,1.47])^d^	IRR 1.23 (95% CI [1.02,1.49])^c^	IRR 1.38 (95% CI [1.14,1.67])^c^	IRR 1.20 (95% CI [0.96,1.50])^c^	parental age; education; smoking; income; countries of origin; cohabitation status; place; family history of psychiatric disorders; asthma	parity; preterm delivery	sex; calendar year of follow-up; congenital malformation; childhood history of asthma or asthma treatment
Gong T. and colleagues (2019) [21]	ASD		OR 1.02 (95% CI [0.85,1.24])^1^^,^^e^				smoking status, age, marital status; education level, BMI	birth year, parity	
Liang H. and colleagues (2017) [18]	ADHD	IRR 1.29 (95% CI [1.20,1.39])^f^	IRR 1.21(95% CI [1.09,1.33])^d^	IRR 1.05 (95% CI [0.87,1.25])^c^	IRR 1.17 (95% CI [1.00,1.38])^c^	IRR 1.48 (95% CI [1.24,1.78])^c^	paternal age at childbirth; maternal age at childbirth; maternal education; maternal smoking during pregnancy; maternal socioeconomic status; parental history of psychiatric disorders; maternal history of asthma before delivery and inhaled glucocorticoid use during pregnancy	birth year; parity	sex
Nagata A. and colleagues (2023) [24]	Communication skills		* Exposure to B2AA: OR 1.33 (95% CI [0.68,2.62])^d^ * Exposure to corticosteroids: OR 1.19 (95% CI [0.80,1.77])^d^	* Exposure to B2AA: OR 0.71 (95% CI [0.23,2.21])^c^ *Exposure to Corticosteroids: OR 1.00 (95% CI [0.59,1.67])^c^			maternal age at delivery; marital status; education; asthma; alcohol consumption during pregnancy; maternal and paternal smoking during pregnancy and household annual income		sex
	Gross motor skills		* Exposure to B2AA: OR 0.79 (95% CI [0.51,1.21])^d^ * Exposure to corticosteroids: OR 1.08 (95% CI [0.82,1.42])^d^	* Exposure to B2AA: OR 0.99 (95% CI [0.56,1.74])^c^ *Exposure to Corticosteroids: OR 0.83 (95% CI [0.59,1.16])^c^			maternal age at delivery; marital status; education; asthma; alcohol consumption during pregnancy; maternal and paternal smoking during pregnancy and household annual income		sex
	Fine motor skills		* Exposure to B2AA: OR 0.81 (95% CI [0.51,1.28])^d^ * Exposure to corticosteroids: OR 1.18 (95% CI [0.92,1.50])^d^	* Exposure to B2AA: OR 0.67 (95% CI [0.38,1.17])^c^ *Exposure to Corticosteroids: OR 0.89 (95% CI [0.66,1.22])^c^			maternal age at delivery; marital status; education; asthma; alcohol consumption during pregnancy; maternal and paternal smoking during pregnancy and household annual income		sex
	Problem solving skills		* Exposure to B2AA: OR 1.05 (95% CI [0.69,1.60])^d^ * Exposure to corticosteroids: OR 1.15 (95% CI [0.91,1.46])^d^	* Exposure to B2AA: OR 0.81 (95% CI [0.49,1.35])^c^ *Exposure to Corticosteroids: OR 0.96 (95% CI [0.71,1.29])^c^			maternal age at delivery; marital status; education; asthma; alcohol consumption during pregnancy; maternal and paternal smoking during pregnancy and household annual income		sex
	Personal-social skills		* Exposure to B2AA: OR 0.52 (95% CI [0.25,1.07])^d^ * Exposure to corticosteroids: OR 0.89 (95% CI [0.56,1.41])^d^	* Exposure to B2AA: OR 0.68 (95% CI [0.24,1.95])^c^ *Exposure to Corticosteroids: OR 0.93 (95% CI [0.57,1.52])^c^			maternal age at delivery; marital status; education; asthma; alcohol consumption during pregnancy; maternal and paternal smoking during pregnancy and household annual income		sex
Li L. and colleagues (2018) [23]	Cerebral palsy	OR 1.06 (95% CI [0.82,1.39])^g^	OR 1.10 (95% CI [0.78,1.53])^d^				maternal age at delivery; paternal age; maternal education; maternal cohabitation status; maternal smoking status; maternal history of Cerebral Palsy; maternal asthma hospital-diagnosed before delivery	birth year; parity	sex
Kemppainen M. and colleagues (2024) [39]	ADHD		HR 1.54 (95% CI [1.44,1.65])^h^				maternal age, socioeconomic status	birth year; parity	
	ASD		HR 1.42 (95% CI [1.28,1.58])^h^				maternal age, socioeconomic status	birth year; parity	
	Motor developmental disorder		HR 1.48 (95% CI [1.33,1.65])^h^				maternal age, socioeconomic status	birth year; parity	
	Learning disabilities		HR 1.62 (95% CI [1.40,1.87])^h^				maternal age, socioeconomic status	birth year; parity	
	Language developmental disorder		HR 1.17 (95% CI [1.08,1.26])^h^				maternal age, socioeconomic status	birth year; parity	
	Mixed developmental disorder		HR 1.26 (95% CI [1.13,1.41])^h^				maternal age, socioeconomic status	birth year; parity	
	Intellectual disability		HR 1.18 (95% CI [1.03,1.36])^h^				maternal age, socioeconomic status	birth year; parity	

ASD, autism spectrum disorder; ADHD, attention-deficit hyperactivity disorder; BMI, body mass index; B2AA, beta-2 adrenergic agonists; CI, confidence interval; HR, hazard ratio, IRR, incidence rate ratio; OR odd ratio.

^a^Reference group had no exposure to any B2AA or mimics from 30 days before conception through delivery.

^b^Reference group had no exposure during any exposure period.

^c^Reference group had no exposure to B2AA from one year prior to and during pregnancy.

^d^Reference group had no exposure to B2AA during pregnancy.

^e^Reference group had asthma but with no medications.

^f^Reference group had no exposure to B2AA from 2 years before pregnancy through delivery.

^g^Reference group had never used B2AA.

^h^Reference group had no asthma and were not using any asthma medication from 3 months prior to pregnancy until delivery.

^1^Inhaled B2AA with or without other asthma medications.

### Timing and duration of exposure

Three studies showed significant associations for exposure any time during pregnancy (two for ASD; one for ADHD) and reported significant associations with preconception exposure and exposure specifically during the second trimester [18–20] (Table 2). Two studies reported significant associations with third-trimester exposure, and one study found significant associations with first-trimester exposure (Table 2). One study, that conducted additional analyses examining the duration of exposure, reported that there was an association between second-trimester B2AA exposure and ASD for exposures lasting 45 days or longer (aOR 1.5, 95% CI [1.1,2.0]) but not for shorter exposures (aOR 1.2, 95% CI [0.9,1.6]) [20]. Although the point estimate was higher for longer duration exposure, which could be consistent with a possible dose-response pattern, the confidence intervals overlapped, indicating limited evidence of a meaningful difference in magnitude between the two exposure categories.

### Stratification for maternal asthma and offspring sex

Five of the eight studies used statistical adjustment and/or sub-group analyses to differentiate associations with asthma medication from associations with asthma [18–21,24] (Table D in S1 Appendix). The five studies reported non-significant associations between B2AA and ASD [19–21], ADHD [18], and other neurodevelopmental problems (communication, gross motor, fine motor, problem solving, and personal-social) [24] in the sub-group of mothers with confirmed asthma. One of the studies that reported sub-group analyses found a significant association between asthma medication and offspring ASD in women without asthma diagnoses [19]. In the one study that used statistical adjustment for maternal asthma, the association between third-trimester exposure to B2AA and ASD was significant when adjusted for maternal asthma [20].

Four studies reported sex-stratified analyses to take account of potential effect modification by the offspring’s sex [18,19,23,24] (Table E in S1 Appendix). The only study of ASD to do sex sub-group analyses reported that the association between B2AA exposure at some point during pregnancy and ASD was significant in both boys and girls [19]. In contrast, whilst preconception exposure to B2AA was associated with ADHD in both boys and girls, exposure during pregnancy was only associated with ADHD in boys [18]. However, the confidence intervals overlapped, so there was no definitive evidence of a difference in the magnitude of the association by sex. Associations with cerebral palsy were non-significant in both boys and girls [23], and no modifying effect of sex was reported in relation to communication, gross and fine motor problems, problem solving, and personal-social difficulties [24].

### Meta-analyses

**Fig 2:**
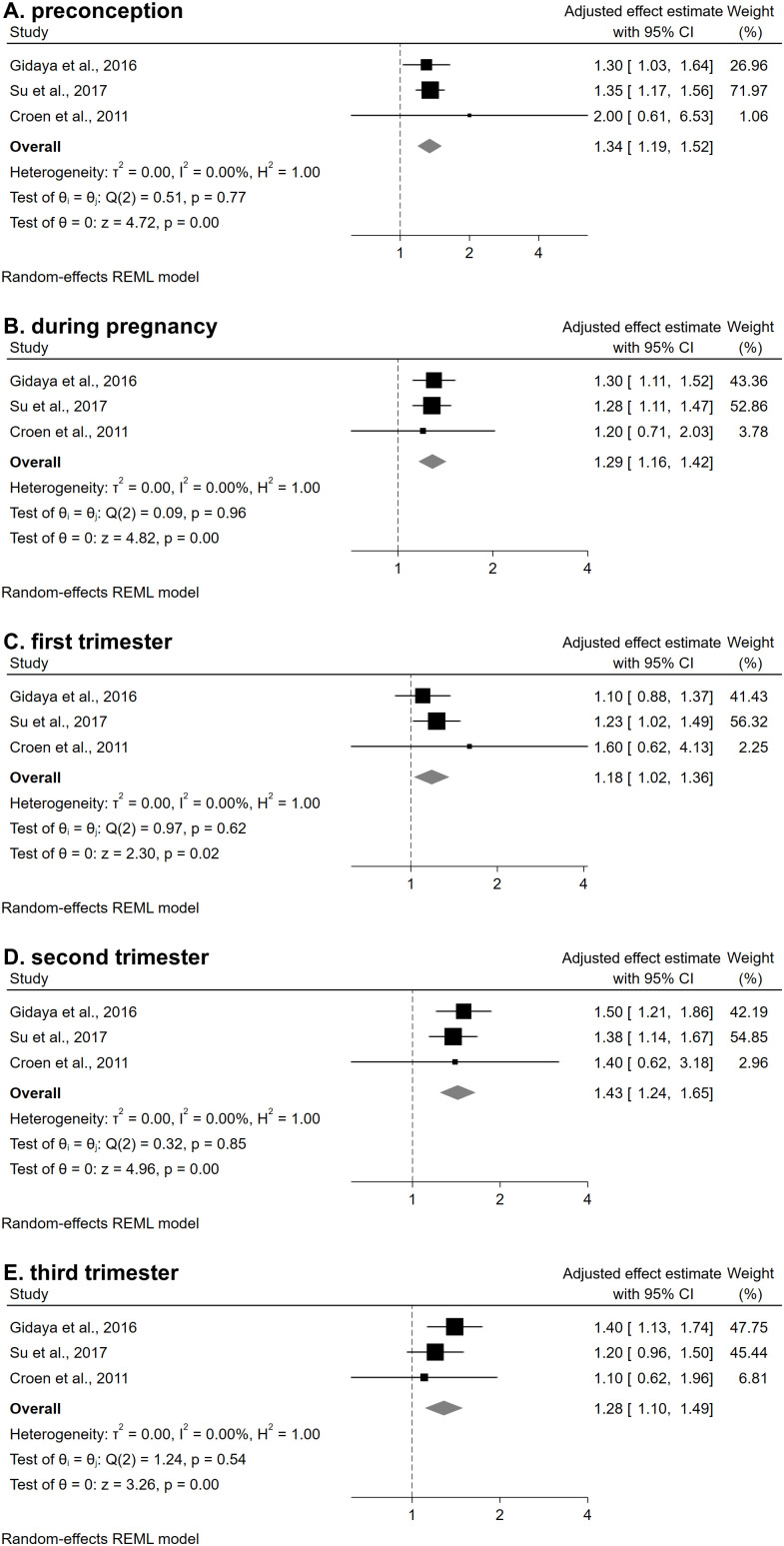
Forest plots for each random effects meta-analysis by trimester of pregnancy: (A) preconception, (B) during pregnancy, (C) first trimester, (D) second trimester, and (E) third trimester. Effect estimates are adjusted measures reported by each study: IRR, incidence rate ratio; OR, odds ratio. Su and colleagues reported IRRs, whereas Croen and colleagues and Gidaya and colleagues reported ORs.

Meta-analysis was performed on the three studies that investigated the same exposure (B2AA) and outcome (ASD) [19,20,22], with a combined sample size of 1,380,871 participants. We conducted five separate meta-analyses based on timing of exposure: preconception, any time during pregnancy, and exposure during each trimester. Statistically significant associations were observed between B2AA exposure and ASD for all exposure time periods examined. The magnitude of association varied by timing of exposure. The largest pooled effect estimate was associated with exposure during the second trimester (aOR 1.43, 95% CI [1.24,1.65]), followed by the preconception period (aOR 1.34, 95% CI [1.19,1.52]), any time during pregnancy (aOR 1.29, 95% CI [1.16,1.42]), third trimester (aOR 1.28, 95% CI [1.10,1.49]), and first trimester (aOR 1.18, 95% CI [1.02,1.36]) (Fig 2).

**Fig 3:**
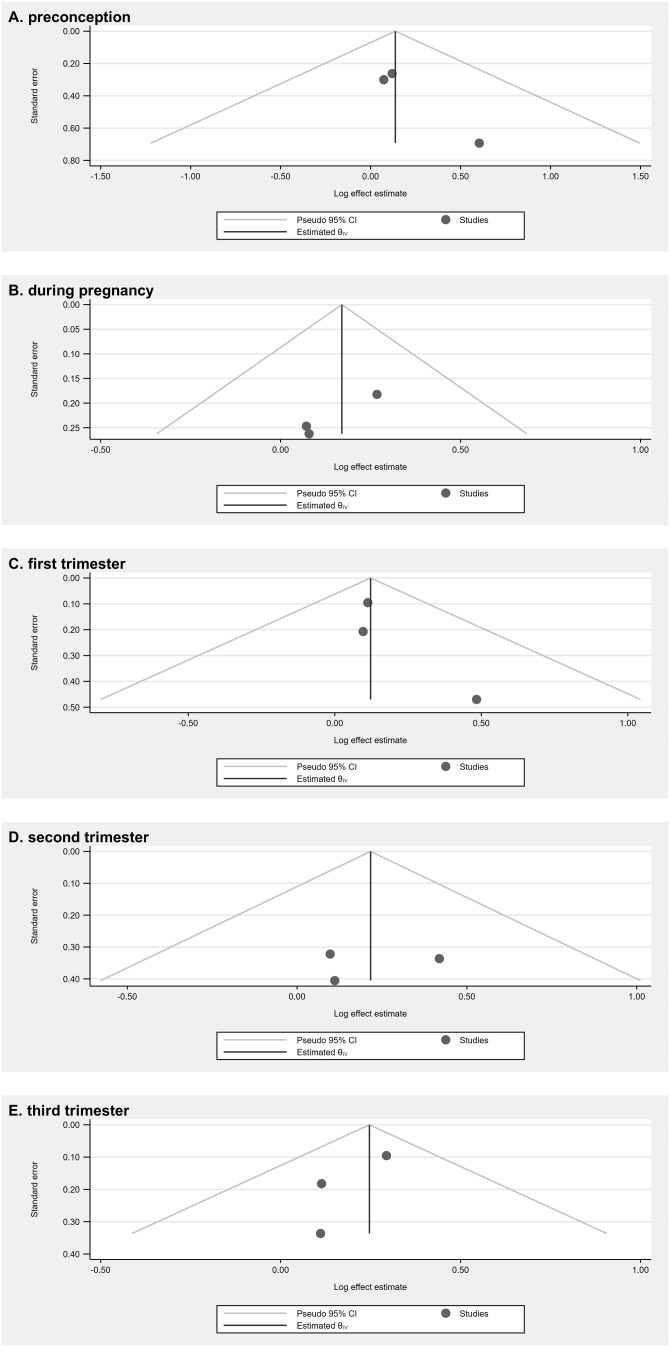
Funnel plots for each random effects meta-analysis by trimester of pregnancy: (A) preconception, (B) during pregnancy, (C) first trimester, (D) second trimester, and (E) third trimester.

In all analyses, the funnel plots appeared symmetrical around the overall-effect line (Fig 3). Egger’s regression tests and Cochran’s Q tests were non-significant. However, given the limited number of included studies, these tests may be unreliable and underpowered, which could lead to a failure to detect true asymmetry. Furthermore, although the *I*² values suggested low heterogeneity, the confidence intervals were wide and included values above 50% indicating that substantial heterogeneity cannot be excluded (Table F in S1 Appendix).

Removing one study [22], in the sensitivity analysis, did not substantially affect the pooled effect estimates. The association between B2AA and ASD remained significant for exposure preconception, any time during pregnancy, and in the third trimester specifically (Figs A and B in S1 Appendix). Furthermore, using a fixed-effects model produced the same results as the random-effects model (Table G, Figs C and D in S1 Appendix).

## Discussion

Studies of prenatal exposure to asthma medication and neurodevelopmental outcomes are few in number and have primarily focused on B2AA exposure and ASD. Meta-analysis of these studies revealed consistent associations between both preconception and prenatal B2AA exposure and ASD, with similar effect sizes across different exposure periods and low statistical heterogeneity. However, the existing evidence is not conclusive as to whether the association is with asthma treatment or asthma, due to confounding by indication. Evidence of an association with ADHD is based on a single study, which suggested that any effect of in-utero exposure to medication may be specific to boys. However, these findings require corroboration because, in addition to the limited number of studies meta-analyzed (*n* = 3), the continuous usage of B2AA, for more than 45 days during each stage of pregnancy, might indicate more severe or uncontrolled asthma, reflecting confounding by severity. To date, based on our search strategy, no associations have been demonstrated with other neurodevelopmental outcomes: gross and fine motor skills, communication and problem-solving skills, and cerebral palsy.

Previous research on the safety of B2AA use during pregnancy has predominantly focused on immediate maternal and pregnancy outcomes, rather than longer-term neurodevelopmental effects. A narrative systematic review of early outcomes reported associations between congenital anomalies and prenatal exposure to both short-acting (SABA) and long-acting (LABA) beta-2-adrenergic agonist. However, these findings were limited by use of comparison groups that included, or were restricted to, women without asthma, making it impossible to differentiate between the effect of B2AA medication and the effect of the underlying condition [7]. Two subsequent narrative reviews reached different conclusions, finding no convincing evidence of early adverse outcomes associated with SABAs use during pregnancy and, specifically, no evidence of an association with structural birth defects [40,41] and concluded that SABAs should be used as the first-line medication during pregnancy. However, one of the reviews also highlighted the scarcity of data on LABAs [40], and recommended that LABAs should only be used as an adjuvant therapy if asthma cannot be well controlled by moderate doses of ICS.

Our review adds new evidence to previous reviews by focusing on longer-term neurodevelopmental outcomes. The methodology was robust; we used a PECO framework, adhered to the PRISMA guidelines, and assessed the quality of included studies. Several limitations of the included studies may have influenced their results. Mothers were assumed to have actually taken their asthma medications following collection, leading to the possibility of exposure misclassification [20,21,23,24]. Use of secondary data, predominantly from registries meant that investigators were unable to adjust for unmeasured covariates, leading to potential residual confounding [20,24]. For example, one study cited their lack of data on environmental exposures that could contribute to both maternal asthma and childhood outcomes [20]. Furthermore, only two studies controlled for parental history of psychiatric disorders [18,19] which may act as a confounder because of the inherited nature of autism [42,43], and poorer asthma control and more frequent exacerbations among mothers with anxiety or depression [44,45]. Notably, only two studies used a directed acyclic graph (DAG) to inform their selection of potential confounders [21,24]. Moreover, some studies were limited by incomplete coverage of registers over the follow-up period [21] and some had incomplete ascertainment of outcomes due to the registries not including all healthcare providers [18,19]. Also, the Danish National Cerebral Palsy Register records cerebral palsy at one year of age resulting in misclassification of children with cerebral palsy who die in the first year of life [23]. Furthermore, some neurodevelopmental disorders may not be diagnosed until adolescence or adulthood, resulting in incomplete ascertainment over the follow-up period [39]. Where studies used registries, data were often missing on the severity of maternal asthma and the dose, duration and route of administration of asthma medications [24] and, in studies that collected data through interviews, recall bias was possible [24]. More generally, it is not possible to determine causality using observational data [21].

Drawing a conclusive result on the association between B2AA and neurodevelopmental disorders is challenging, due to several important limitations and knowledge gaps in the existing literature.

A key limitation is that the included studies examined B2AA exposure without adequately accounting for concomitant use of other asthma medications or the severity of maternal asthma. Since asthma management typically follows a stepwise approach, women with more severe symptoms likely used multiple medications simultaneously. Moreover, although the use of B2AA as monotherapy may reflect mild asthma in some individuals, others with moderate to severe asthma may also rely on B2AA as their primary treatment because it is less expensive than controller therapies. Only three studies accounted for potential socioeconomic confounders (e.g., income) [18,19,24]. Importantly, none of the included studies reported whether B2AA was actually administered as a monotherapy or in combination with controller therapy, limiting our ability to determine whether observed associations reflected medication effects, underlying asthma severity, or socioeconomic confounding. Additionally, regarding potential confounding by indication, Gidaya and colleagues reported no substantial change after adjusting for maternal asthma, whereas Su and colleagues observed changes in results when stratifying by maternal asthma status, suggesting confounding by indication [19,20]. Although both studies adjusted for maternal asthma, we were unable to rule out the possibility of residual confounding by indication in our meta-analysis.

The studies did not differentiate between short-acting (SABA) and long-acting (LABA) beta-2-adrenergic agonists, which have important pharmacological differences despite sharing the same basic mechanism of action. SABAs act rapidly (within 5 min) with the effects lasting up to approximately 4 hours [46], and are typically used, as needed, for acute symptom relief. In contrast, LABAs have variable onset times (from 3 to 5 min in the case of formoterol, indacaterol, and olodaterol, compared with up to 20 min for salmeterol [47]), with effects persisting for at least 12 hours, making them suitable for maintenance therapy [46,47]. These distinct usage patterns could theoretically result in different fetal exposure profiles and potentially different neurodevelopmental effects. The inability to distinguish between these medication subtypes in the current literature represents a significant limitation. Moreover, information on medication dosage and, with one exception, treatment duration was missing, which limited our ability to fully assess the broader picture of prenatal exposure to asthma medications. Similarly, the included studies had a narrow scope, concentrating mainly on B2AA, and to a lesser extent, ICS. This constrained our assessment, even though our comprehensive search strategy was designed to capture evidence on all asthma medications.

Another important gap is the limited geographical representation of the available evidence. All included studies were conducted in high-income countries with well-established healthcare systems and, in some cases like Denmark, universal healthcare access [18,23]. The absence of data from low- and middle-income countries raises concerns about the global generalizability of these findings, particularly in settings where healthcare access, medication availability, and asthma management practices may differ substantially. Studies including more diverse populations with varied sociodemographic characteristics and healthcare contexts would provide more globally representative evidence.

Finally, it is important not to rely solely on p-values when interpreting statistical significance. For example, although Gidaya and colleagues and Su and colleagues reported associations of approximately similar magnitude between prenatal exposure to B2AA and ASD, the wider confidence interval reported by Su and colleagues reflected greater uncertainty around the estimate. In addition, because we examined several exposure windows in our meta-analysis, multiple comparisons may have increased the likelihood of chance findings. Each additional test raises the probability of identifying a statistically significant association purely by chance, thereby increasing the risk of false-positive results.

Our meta-analyses suggested an association between ASD and B2AA exposure across all examined exposure periods, including preconception, during pregnancy, and each individual trimester. Further corroboration is required given that the ASD meta-analyses were based on only three studies and that ADHD has only been investigated in a single study. The general consistent direction of the meta-analyses findings, together with the high study quality scores, low heterogeneity, and lack of clear evidence of bias, provide cautious support for the observed association. B2AA medications are often used alongside other asthma treatments as part of comprehensive management strategies. According to the GINA guidelines, ICS-formoterol (a LABA combined with ICS) is recommended as both the reliever therapy and as a maintenance-and-reliever therapy (MART) in Track 1. In Track 2, patients use a SABA as the reliever, taken either alone or together with ICS, depending on whether they are receiving ICS alone or ICS-LABA as a maintenance therapy. Therefore, future research should better account for polypharmacy, asthma severity, and specific B2AA subtypes (short-acting versus long-acting) in order to strengthen the evidence base and better inform clinical decision-making regarding asthma medication use during pregnancy.

## Supporting information

S1 Appendix **Table A.** Search strategy using PubMed, Medline and Embase. **Table B.** Assessment of methodological quality using the Newcastle-Ottawa Scale. **Table C.** Medication exposure investigated in studies. **Table D.** Sub-group analyses of the associations between beta-2-adrenergic agonists and neurodevelopmental outcomes stratified by maternal asthma diagnosis. **Table E.** Sub-group analyses of the associations between beta-2-adrenergic agonists and autism spectrum disorder stratified by child’s sex. **Table F.** Test of heterogeneity and publication bias. **Table G.** Test of heterogeneity and publication bias for the fixed-effects model. **Fig A.** Forest plots for each random effects meta-analysis by trimester of pregnancy after excluding study by Croen and colleagues. **Fig B.** Funnel plots for each random effects meta-analysis by trimester of pregnancy after excluding study by Croen and colleagues. **Fig C.** Forest plots for each fixed effects meta-analysis by trimester of pregnancy. **Fig D.** Funnel plots for each fixed effects meta-analysis by trimester of pregnancy. **Checklist A**. PRISMA 2020 Checklist [48]. Page MJ, McKenzie JE, Bossuyt PM, Boutron I, Hoffmann TC, Mulrow CD, et al. The PRISMA 2020 statement: an updated guideline for reporting systematic reviews. 2021. https://doi.org/10.1136/bmj.n71. This checklist is licenced under the Creative Commons Attribution 4.0 International License (CC BY 4.0; https://creativecommons.org/licenses/by/4.0/).
(DOCX)

## Abbreviations

ACS: antenatal corticosteroids
ADHD: attention-deficit hyperactivity disorder
ASD: autism spectrum disorder
ATC: Anatomical Therapeutic Chemical
B2AA: beta-2-adrenergic agonists
CIs: confidence intervals
DAG: directed acyclic graph
GINA: Global Initiative for Asthma
ICD-11: 11th Revision of the International Classification of Diseases
ICS: inhaled corticosteroids
ICS-LABA: ICS-long-acting beta-2-adrenergic agonist
LTRA: leukotriene antagonist
MART: maintenance-and-reliever therapy
MCS: mast cell stabilizers
NICE: National Institute for Health and Care Excellence
OCS: oral corticosteroids
PECO: Population Exposure Comparator Outcome
PRISMA: Preferred Reporting Items for Systematic reviews and Meta-Analyses
REML: restricted maximum likelihood
SABA: short-acting beta-2-adrenergic agonist
SEN: special educational needs
WHO: World Health Organization
