# Supplementary material for: Prenatal exposure to asthma medications and risk of neurodevelopmental disorders and educational difficulties: A systematic review and meta-analysis
Source: PLoS Med. 2026 Jun 1;23(6):e1005100. doi: 10.1371/journal.pmed.1005100 (PMC13225639; doi:10.1371/journal.pmed.1005100)
Supplement: S1 Appendix — Table A. Search strategy using PubMed, Medline and Embase. Table B. Assessment of methodological quality using the Newcastle-Ottawa Scale. Table C. Medication exposure investigated in studies. Table D. Sub-group analyses of the associations between beta-2-adrenergic agonists and neurodevelopmental outcomes stratified by maternal asthma diagnosis. Table E. Sub-group analyses of the associations between beta-2-adrenergic agonists and autism spectrum disorder stratified by child’s sex. Table F. Test of heterogeneity and publication bias. Table G. Test of heterogeneity and publication bias for the fixed-effects model. Fig A. Forest plots for each random effects meta-analysis by trimester of pregnancy after excluding study by Croen and colleagues. Fig B. Funnel plots for each random effects meta-analysis by trimester of pregnancy after excluding study by Croen and colleagues. Fig C. Forest plots for each fixed effects meta-analysis by trimester of pregnancy. Fig D. Funnel plots for each fixed effects meta-analysis by trimester of pregnancy. Checklist A. PRISMA 2020 Checklist [48]. Page MJ, McKenzie JE, Bossuyt PM, Boutron I, Hoffmann TC, Mulrow CD, et al. The PRISMA 2020 statement: an updated guideline for reporting systematic reviews. 2021. https://doi.org/10.1136/bmj.n71. This checklist is licenced under the Creative Commons Attribution 4.0 International License (CC BY 4.0; https://creativecommons.org/licenses/by/4.0/). (DOCX) [file pmed.1005100.s001.docx]

**S1 Appendix**

Table of Contents

[**Table A.** Search strategy using PubMed, Medline and Embase 1](#_Toc229402272)

[**Table B.** Assessment of methodological quality using the Newcastle-Ottawa Scale 13](#_Toc229402273)

[**Table C.** Medication exposure investigated in studies 15](#_Toc229402274)

[**Table D.** Sub-group analyses of the associations between beta-2-adrenergic agonists and neurodevelopmental outcomes stratified by maternal asthma diagnosis 16](#_Toc229402275)

[**Table E.** Sub-group analyses of the associations between beta-2-adrenergic agonists and autism spectrum disorder stratified by child’s sex 33](#_Toc229402276)

[**Table F.** Test of heterogeneity and publication bias 47](#_Toc229402277)

[**Table G.** Test of heterogeneity and publication bias for the fixed-effects model 47](#_Toc229402278)

[**Fig A.** Forest plots for each random effects meta-analysis by trimester of pregnancy after excluding study by Croen et al.* 48](#_Toc229402279)

[**Fig B.** Funnel plots for each random effects meta-analysis by trimester of pregnancy after excluding study by Croen et al. 51](#_Toc229402280)

[**Fig C.** Forest plots for each fixed effects meta-analysis by trimester of pregnancy* 54](#_Toc229402281)

[**Fig D.** Funnel plots for each fixed effects meta-analysis by trimester of pregnancy 56](#_Toc229402282)

[**Checklist A.** PRISMA 2020 Checklist 59](#_Toc229402283)

# **Table A.** Search strategy using PubMed, Medline and Embase

*PubMed*
**((((((((pregnancy[mh:noexp]) OR ((((pregnancy trimesters[mh:noexp]) OR (pregnancy trimester, first[mh:noexp]) OR (pregnancy trimester, second[mh:noexp])) OR (pregnancy trimester, third[mh:noexp])))) OR (prenatal exposure delayed effects[mh:noexp])) OR (pregnan*[Text Word] OR "in-utero"[Text Word] OR utero[Text Word] OR uterus[Text Word] OR "intra-uterine"[Text Word] OR intrauterine[Text Word] OR prenatal[Text Word] OR "pre-natal"[Text Word] OR antenatal[Text Word] OR "ante-natal"[Text Word] OR perinatal[Text Word] OR maternal[Text Word])) OR (gestation[Title/Abstract])) AND ((((child, preschool[mh:noexp] OR child[mh:noexp]) OR (infants, newborn[mh:noexp] OR infant[mh:noexp] OR adolescent[mh:noexp])) OR (fetus[mh:noexp])) OR (toddler*[Text Word] OR infant*[Text Word] OR neonat*[Text Word] OR fetus[Text Word] OR foetus[Text Word] OR foetal[Text Word] OR fetal[Text Word] OR baby[Text Word] OR babies[Text Word] OR offspring*[Text Word] OR kindergarten*[Text Word] OR school children[Text Word] OR schoolchildren[Text Word] OR school-children[Text Word] OR school pupil[Text Word] OR school pupils[Text Word] OR adolescen*[Text Word] OR teen*[Text Word] OR youth[Text Word] OR school[Text Word]))) AND ((((((((((((((((((((((((((((((((((((((((((((((Anti-Asthmatic Agents[MeSH Terms]) OR (Adrenergic beta-2 Receptor Agonists[MeSH Terms])) OR (Bronchodilator Agents[mh:noexp])) OR (albuterol[MeSH Terms])) OR (Albuterol*[Title/Abstract])) OR (albuterol[Text Word] OR salbutamol[Text Word])) OR (levalbuterol[MeSH Terms])) OR (Levalbuterol*[Title/Abstract])) OR (Terbutaline[MeSH Terms])) OR (Terbutaline*[Title/Abstract])) OR (Salmeterol Xinafoate[MeSH Terms])) OR (Salmeterol*[Title/Abstract])) OR (Formoterol Fumarate[MeSH Terms])) OR (Formoterol*[Title/Abstract])) OR ("long* acting beta* agonist"[Text Word] OR "short* acting beta* agonist*"[Text Word] OR SABA[Text Word] OR LABA[Text Word] OR albuterol[Text Word] OR salbutamol[Text Word] OR levalbuterol[Text Word] OR terbutaline[Text Word] OR salmeterol[Text Word] OR formoterol[Text Word])) OR (Steroids[MeSH Terms])) OR (dexamethasone[MeSH Terms])) OR (cortico*[Text Word])) OR (dexamethasone*[Title/Abstract])) OR (methylprednisolone[MeSH Terms])) OR (Methylprednisolone*[Title/Abstract])) OR (prednisolone[MeSH Terms])) OR (Prednisolone*[Title/Abstract])) OR (dexamethasone[Text Word] OR methylprednisolone[Text Word] OR prednisolone[Text Word] OR prednisone[Text Word])) OR (Fluticasone[MeSH Terms])) OR (Fluticasone*[Title/Abstract])) OR (budesonide[MeSH Terms])) OR (Budesonide*[Title/Abstract])) OR (Mometasone Furoate[MeSH Terms])) OR (Mometasone*[Title/Abstract])) OR (Beclomethasone[MeSH Terms])) OR (Beclomethasone*[Title/Abstract])) OR (Ciclesonide*[Title/Abstract])) OR (fluticasone[Text Word] OR budesonide[Text Word] OR mometasone[Text Word] OR beclomethasone[Text Word] OR ciclesonide[Text Word])) OR (Cromolyn Sodium[MeSH Terms])) OR (sodium cromoglycate[Text Word] OR cromolyn sodium[Text Word])) OR (Nedocromil[MeSH Terms])) OR (nedocromil sodium[Text Word] OR nedocromil[Text Word])) OR ("nebulizers and vaporizers"[mh:noexp])) OR (dry powder inhalers[mh:noexp])) OR (metered dose inhalers[mh:noexp])) OR (Theophylline[MeSH Terms])) OR (Theophylline*[Title/Abstract])) OR (leukotriene antagonists[MeSH Terms])) OR (Leukotriene Antagonist*[Title/Abstract])) OR (montelukast[Text Word] OR zafirlukast[Text Word] OR zileuton[Text Word]))) AND ((((((((((((((Neurodevelopmental Disorders[MeSH Terms]) OR (((autism spectrum disorder[mh:noexp]) OR (asperger syndrome[mh:noexp])) OR (autistic disorder[mh:noexp]))) OR (attention deficit disorder with hyperactivity[mh:noexp])) OR (Motor Disorders[mh:noexp])) OR (intellectual disability[mh:noexp])) OR (Learning Disabilities[mh:noexp])) OR (child development[mh:noexp])) OR (Developmental Disabilities[mh:noexp])) OR ("child development*"[Text Word])) OR (executive function[mh:noexp])) OR (Cognitive Dysfunction[mh:noexp])) OR ("Executive function*"[Text Word])) OR (((education, special[mh:noexp]) OR (education of hearing disabled[mh:noexp])) OR (education of intellectually disabled[mh:noexp]))) OR (neurodevelopment*[Text Word] OR "neurodevelopmental disorder*"[Text Word] OR "educational difficult*"[Text Word] OR "mental deficienc*"[Text Word] OR "cognitive deficit*"[Text Word] OR "poor performance"[Text Word] OR behavio*[Text Word] OR "behavioural disturbance*"[Text Word] OR social*[Text Word] OR emotion*[Text Word] OR "motor impairment*"[Text Word] OR communication*[Text Word] OR "learning disabilit*"[Text Word] OR "learning difficult*"[Text Word] OR "learning disorder*"[Text Word] OR autism[Text Word] OR "intellectual disabilit*"[Text Word] OR "intellectual impairment*"[Text Word] OR "mental retardation*"[Text Word] OR "attention deficit hyperactivity disorder*"[Text Word] OR "attention deficit disorder*"[Text Word] OR ADHD[Text Word] OR "cerebral palsy"[Text Word] OR "cognitive impairment*"[Text Word] OR "cognitive disabilit*"[Text Word] OR "developmental disorder*"[Text Word] OR "developmental disabilit*"[Text Word] OR "development disabilit*"[Text Word] OR "cognitive disturbance*"[Text Word] OR "autism spectrum"[Text Word] OR autistic[Text Word] OR "autism spectrum disorder*"[Text Word] OR Asperger[Text Word] OR Asperger's[Text Word] OR "Asperger's Syndrome"[Text Word] OR hyperactiv*[Text Word] OR overactive*[Text Word] OR inattention[Text Word] OR "hyperkinetic disorder*"[Text Word] OR hyperkinet*[Text Word] OR "communication disorder*"[Text Word] OR "motor disorder*"[Text Word] OR "school performance"[Text Word] OR "additional education*"[Text Word] OR "additional educational need*"[Text Word] OR "additional support need*"[Text Word] OR "assisted support"[Text Word] OR "academic attainment*"[Text Word]))) NOT (animals[mh:noexp]) Filters: English, from 2003 – 2024**

*Medline*

| **#** | **Query** |
| --- | --- |
| 1 | Pregnancy/ |
| 2 | pregnancy trimesters/ or pregnancy trimester, first/ or pregnancy trimester, second/ or pregnancy trimester, third/ |
| 3 | Prenatal Exposure Delayed Effects/ |
| 4 | (pregnan$ or in-utero or utero or uterus or intra-uterine or intrauterine or prenatal or pre-natal or antenatal or ante-natal or perinatal or maternal).mp. |
| 5 | [gestation.tw](http://gestation.tw/). |
| 6 | or/1-5 |
| 7 | child/ or child, preschool/ |
| 8 | infant/ or infant, newborn/ |
| 9 | Fetus/ |
| 10 | Adolescent/ |
| 11 | (toddler* or infant* or neonat* or fetus or foetus or foetal or fetal or baby or babies or offspring* or kindergarten* or school children or school-children or schoolchildren or school pupil or school pupils or school* or teen* or adolescen* or youth).mp. |
| 12 | or/7-11 |
| 13 | exp Anti-Asthmatic Agents/ |
| 14 | exp Adrenergic beta-2 Receptor Agonists/ |
| 15 | Bronchodilator Agents/ |
| 16 | exp Albuterol/ |
| 17 | Albuterol$.tw. |
| 18 | (albuterol or salbutamol).mp. |
| 19 | exp Levalbuterol/ |
| 20 | Levalbuterol$.tw. |
| 21 | exp Terbutaline/ |
| 22 | Terbutaline$.tw. |
| 23 | exp Salmeterol Xinafoate/ |
| 24 | Salmeterol$.tw. |
| 25 | exp Formoterol Fumarate/ |
| 26 | Formoterol$.tw. |
| 27 | (long* acting beta* agonist or short* acting beta* agonist* or SABA or LABA or albuterol or salbutamol or levalbuterol or terbutaline or salmeterol or formoterol).mp. |
| 28 | exp Steroids/ |
| 29 | exp Dexamethasone/ |
| 30 | cortico$.mp. |
| 31 | dexamethasone$.tw. |
| 32 | exp Methylprednisolone/ |
| 33 | Methylprednisolone$.tw. |
| 34 | exp Prednisolone/ |
| 35 | Prednisolone$.tw. |
| 36 | exp Prednisone/ |
| 37 | Prednisone$.tw. |
| 38 | (dexamethasone or methylprednisolone or prednisolone or prednisone).mp. |
| 39 | exp Fluticasone/ |
| 40 | Fluticasone$.tw. |
| 41 | exp Budesonide/ |
| 42 | Budesonide$.tw. |
| 43 | exp Mometasone Furoate/ |
| 44 | Mometasone$.tw. |
| 45 | exp Beclomethasone/ |
| 46 | Beclomethasone$.tw. |
| 47 | Ciclesonide$.tw. |
| 48 | (fluticasone or budesonide or mometasone or beclomethasone or ciclesonide).mp. |
| 49 | exp Cromolyn Sodium/ |
| 50 | (sodium cromoglycate or cromolyn sodium).mp. |
| 51 | exp Nedocromil/ |
| 52 | (nedocromil sodium or nedocromil).mp. |
| 53 | "nebulizers and vaporizers"/ or dry powder inhalers/ or metered dose inhalers/ |
| 54 | exp Theophylline/ |
| 55 | Theophylline$.tw. |
| 56 | exp Leukotriene Antagonists/ |
| 57 | Leukotriene Antagonist$.tw. |
| 58 | (montelukast or zafirlukast or zileuton).mp. |
| 59 | or/13-58 |
| 60 | exp Neurodevelopmental Disorders/ |
| 61 | autism spectrum disorder/ or asperger syndrome/ or autistic disorder/ |
| 62 | Attention Deficit Disorder with Hyperactivity/ |
| 63 | Motor Disorders/ |
| 64 | Intellectual Disability/ |
| 65 | Learning Disabilities/ |
| 66 | Child Development/ |
| 67 | Developmental Disabilities/ |
| 68 | child development$.mp. |
| 69 | Executive Function/ |
| 70 | Cognitive Dysfunction/ |
| 71 | Executive function$.mp. |
| 72 | education, special/ or "education of hearing disabled"/ or "education of intellectually disabled"/ |
| 73 | (neurodevelopment* or neurodevelopmental disorder* or educational difficult* or mental deficienc* or cognitive deficit* or poor performance or behavio* or behavioural disturbance* or social* or emotion* or motor impairment* or communication* or learning disabilit* or learning difficult* or learning disorder* or autism or intellectual disabilit* or intellectual impairment* or mental retardation* or attention deficit hyperactivity disorder* or attention deficit disorder* or ADHD or cerebral palsy or cognitive impairment* or cognitive disabilit* or developmental disorder* or developmental disabilit* or development disabilit* or cognitive disturbance* or autism spectrum or autistic or autism spectrum disorder* or Asperger or Asperger's or Asperger's Syndrome or hyperactiv* or overactive* or inattention or hyperkinetic disorder* or hyperkinet* or communication disorder* or motor disorder* or school performance or additional education* or additional education* need* or additional support need* or assisted support or academic attainment*).mp. |
| 74 | or/60-73 |
| 75 | 6 and 12 and 59 and 74 |
| 76 | exp Books/ |
| 77 | editorial/ |
| 78 | letter/ |
| 79 | Animals/ |
| 80 | Humans/ |
| 81 | 79 not (79 and 80) |
| 82 | or/76-79,81 |
| 83 | 75 not 82 |
| 84 | limit 83 to "remove preprint records" |
| 85 | limit 84 to english language |
| 86 | limit 85 to yr="2003 - 2024" |

*Embase*

| **#** | **Query** |
| --- | --- |
| 1 | pregnancy/ |
| 2 | second trimester pregnancy/ or first trimester pregnancy/ or third trimester pregnancy/ |
| 3 | exp prenatal exposure/ |
| 4 | (pregnan$ or in-utero or utero or uterus or intra-uterine or intrauterine or prenatal or pre-natal or antenatal or ante-natal or perinatal or maternal).mp. |
| 5 | gestation.tw. |
| 6 | or/1-5 |
| 7 | child/ or preschool child/ |
| 8 | infant/ or newborn/ |
| 9 | fetus/ |
| 10 | adolescent/ |
| 11 | (toddler* or infant* or neonat* or fetus or foetus or foetal or fetal or baby or babies or offspring* or kindergarten* or school children or school-children or schoolchildren or school pupil or school pupils or school* or teen* or adolescen* or youth).mp. |
| 12 | or/7-11 |
| 13 | exp antiasthmatic agent/ |
| 14 | exp beta 2 adrenergic receptor stimulating agent/ |
| 15 | bronchodilating agent/ |
| 16 | exp salbutamol/ |
| 17 | Albuterol$.tw. |
| 18 | (albuterol or salbutamol).mp. |
| 19 | exp levalbuterol/ |
| 20 | Levalbuterol$.tw. |
| 21 | exp terbutaline/ |
| 22 | Terbutaline$.tw. |
| 23 | exp salmeterol xinafoate/ |
| 24 | Salmeterol$.tw. |
| 25 | exp formoterol fumarate/ |
| 26 | Formoterol$.tw. |
| 27 | (long* acting beta* agonist or short* acting beta* agonist* or SABA or LABA or albuterol or salbutamol or levalbuterol or terbutaline or salmeterol or formoterol).mp. |
| 28 | exp steroid/ |
| 29 | exp dexamethasone/ |
| 30 | cortico$.mp. |
| 31 | dexamethasone$.tw. |
| 32 | exp methylprednisolone/ |
| 33 | Methylprednisolone$.tw. |
| 34 | exp prednisolone/ |
| 35 | Prednisolone$.tw. |
| 36 | exp prednisone/ |
| 37 | Prednisone$.tw. |
| 38 | (dexamethasone or methylprednisolone or prednisolone or prednisone).mp. |
| 39 | exp fluticasone/ |
| 40 | Fluticasone$.tw. |
| 41 | exp budesonide/ |
| 42 | Budesonide$.tw. |
| 43 | exp mometasone furoate/ |
| 44 | Mometasone$.tw. |
| 45 | exp beclometasone/ |
| 46 | Beclomethasone$.tw. |
| 47 | exp Ciclesonide/ |
| 48 | Ciclesonide$.tw. |
| 49 | (fluticasone or budesonide or mometasone or beclomethasone or ciclesonide).mp. |
| 50 | exp cromoglycate disodium/ |
| 51 | (sodium cromoglycate or cromolyn sodium).mp. |
| 52 | exp nedocromil/ |
| 53 | (nedocromil sodium or nedocromil).mp. |
| 54 | exp nebulizer/ |
| 55 | exp theophylline/ |
| 56 | Theophylline$.tw. |
| 57 | exp leukotriene receptor blocking agent/ |
| 58 | Leukotriene Antagonist$.tw. |
| 59 | (montelukast or zafirlukast or zileuton).mp. |
| 60 | or/13-59 |
| 61 | exp developmental disorder/ |
| 62 | autism/ or asperger syndrome/ |
| 63 | attention deficit hyperactivity disorder/ |
| 64 | motor dysfunction/ |
| 65 | intellectual impairment/ |
| 66 | learning disorder/ |
| 67 | child development/ |
| 68 | developmental disorder/ |
| 69 | child development$.mp. |
| 70 | executive function/ |
| 71 | cognitive defect/ |
| 72 | Executive function$.mp. |
| 73 | special education/ or deaf education/ or "education of intellectually disabled"/ |
| 74 | (neurodevelopment* or neurodevelopmental disorder* or educational difficult* or mental deficienc* or cognitive deficit* or poor performance or behavio* or behavioural disturbance* or social* or emotion* or motor impairment* or communication* or learning disabilit* or learning difficult* or learning disorder* or autism or intellectual disabilit* or intellectual impairment* or mental retardation* or attention deficit hyperactivity disorder* or attention deficit disorder* or ADHD or cerebral palsy or cognitive impairment* or cognitive disabilit* or developmental disorder* or developmental disabilit* or development disabilit* or cognitive disturbance* or autism spectrum or autistic or autism spectrum disorder* or Asperger or Asperger's or Asperger's Syndrome or hyperactiv* or overactive* or inattention or hyperkinetic disorder* or hyperkinet* or communication disorder* or motor disorder* or school performance or additional education* or additional education* need* or additional support need* or assisted support or academic attainment*).mp. |
| 75 | or/61-74 |
| 76 | 6 and 12 and 60 and 75 |
| 77 | book/ |
| 78 | conference abstract/ |
| 79 | "conference review"/ |
| 80 | editorial/ |
| 81 | letter/ |
| 82 | animal/ |
| 83 | human/ |
| 84 | 82 not (82 and 83) |
| 85 | or/77-81,84 |
| 86 | 76 not 85 |
| 87 | limit 86 to "remove preprint records" |
| 88 | limit 87 to english language |
| 89 | limit 88 to yr="2003 - 2024" |

# **Table B.** Assessment of methodological quality using the Newcastle-Ottawa Scale

| **Cohort studies** | | | | | | | | | |
| --- | --- | --- | --- | --- | --- | --- | --- | --- | --- |
| **Authors and years** | **Selection** | | | | **Comparability** | **Outcome** | | | **Quality score** |
|  | **Representa-tiveness of the exposed cohort** | **Selection of the non-exposed cohort** | **Ascertainment of exposure** | **Demonstration that outcome of interest was not present at start of study** | **Comparability of cases and controls based on the design or analysis** | **Assessment of outcome** | **Was follow-up long enough for outcomes to occur** | **Adequacy of follow up of cohorts** |  |
| Liang H. et al. (2017) | * | * | * | * | * | * | * | * | Good quality |
| Su X. et al. (2017) | * | * | * | * | * | * | * | * | Good quality |
| Li L. et al.  (2018) | * | * | * | * | * | * | * | * | Good quality |
| Nagata A. et al. (2023) | * | * | * | * | ** | / | * | * | Good quality |
| Kemppainen M. et al. (2024) | * | * | * | * | * | * | * | / | Good quality |
| **Case-control studies** | | | | | | | | | |
| **Authors and years** | **Selection** | | | | **Comparability** | **Exposure** | | | **Quality score** |
|  | **Is the case definition adequate?** | **Representativeness of the cases** | **Selection of Controls** | **Definition of Controls** | **Comparability of cases and controls based on the design or analysis** | **Ascertainment of exposure** | **Same method of ascertainment for cases and controls** | **Non-Response rate** |  |
| Croen L. et al. (2011) | / | / | * | * | ** | * | * | / | Fair quality |
| Gidaya N. et al. (2016) | / | * | * | / | ** | * | * | / | Fair quality |
| Gong T. et al. (2019) | * | * | * | / | * | * | * | / | Good quality |

**Good quality**: 3 or 4 stars in selection domain AND 1 or 2 stars in comparability domain AND 2 or 3 stars in outcome/exposure domain.

**Fair quality**: 2 stars in selection domain AND 1 or 2 stars in comparability domain AND 2 or 3 stars in outcome/exposure domain.

**Poor quality**: 0 or 1 star in selection domain OR 0 stars in comparability domain OR 0 or 1 stars in outcome/exposure domain.

* 1 star

** 2 stars

/ 0 star

# **Table C.** Medication exposure investigated in studies

| **Study** | **Class of asthma medication** | **Anatomical Therapeutic Classification (ATC) codes/ Medications’ names** |
| --- | --- | --- |
| Croen L. et al.(2011) | B2AA | Advair, albuterol, Combivent, Foradil, Maxair, metaproterenol, Ritodrine®, Serevent, terbutaline, Tornalate, and Xopenex. |
| Su X. et al. (2017) | B2AA | R03AC or R03CC |
| Gong T. et al. (2019) | B2AA | R03AC |
|  | ICS | R03BA |
|  | Fixed B2AA-ICS combinations | R03AK |
|  | Oral and injection B2AA | R03CC |
|  | LTRA | R03DC |
|  | Oral corticosteroids | H02AB |
| Liang H. et al. (2017) | B2AA | R03AC or R03CC |
| Nagata A. et al. (2023) | B2AA | R03AC or R03CC |
|  | Corticosteroids | R01AD |
| Li L. et al. (2018) | B2AA | R03AC or R03CC |
| Gidaya N. et al. (2016) | B2AA | R03AC02, R03AC03, R03AC04, R03AC05, R03AC12, R03AC13, R03CC02, R03CC03, and R03CC12 |
| Kemppainen M. et al. (2024) | SABA | R03AC02-3 |
|  | LABA | R03AC04-19 |
|  | ICS | R03BA |
|  | Ciclesonide | R03BA08 |
|  | Short- and long-acting anticholinergics | R03BB |
|  | Combination of LABA and corticosteroids | R03AK07 |
|  | LTRA | R03DC |
|  | Oral corticosteroids | H02AB |
|  | Theophylline | R03DA |
|  | Monohaler therapy of formoterol and budesonide | R03AK07, no R03AC02-3, no R03AC04-19, no R03BA, no other R03AK |

B2AA beta-2-adrenergic agonists, ICS inhaled corticosteroid, LABA long-acting beta-2-adrenergic agonist, LTRA Leukotriene receptor antagonist, SABA short-acting beta-2-adrenergic agonist

# **Table D.** Sub-group analyses of the associations between beta-2-adrenergic agonists and neurodevelopmental outcomes stratified by maternal asthma diagnosis

| Authors and years | Status of maternal asthma | Neurodev-lopmental outcomes | Preconception | During pregnancy | 1st Trimester | 2nd Trimester | 3rd Trimester | | Covariates in the adjusted models | |
| --- | --- | --- | --- | --- | --- | --- | --- | --- | --- | --- |
| Gidaya N. et al. (2016) | Asthmatic | ASD | OR 1.2  (95% CI [0.7,2.1])^a^ | OR 1.4  (95% CI [0.9,2.3])^a^ | OR 1.3  (95% CI [0.8,2.2])^a^ | OR 1.6  (95% CI [0.9,2.7])^a^ | OR 1.7  (95% CI [1.0,2.9])^a^ | | Age, asthma, birth year and month and sex | |
| Gong T. et al. (2019) | Asthmatic | ASD |  | OR 1.02  (95% CI [0.85,1.24])^1,b^ |  |  |  | | Smoking status, age, marital status, education level, BMI, birth year and parity | |
|  | Asthmatic | ASD |  | OR 1.01 (95% CI [0.83,1.22])^1,b^ |  |  |  | | Parity, maternal smoking during pregnancy and civil status at year of child birth, country of birth and age at child birth for mothers and fathers, highest education between parents, maternal BMI at first antenatal visit, pregnancy and delivery risk factors (pre-eclampsia, pre-gestational and gestational diabetes, placental abruption, premature contraction, premature rupture of membranes, hemorrhage during pregnancy/after delivery, gestational age, birth weight, mode of delivery, and parity), as well as 12-month history of asthma exacerbation before pregnancy | |
| Su X. et al. (2017) | Asthmatic | ASD |  | IRR 1.11  (95% CI [0.77,1.59])^c^ | IRR 1.18  (95% CI [0.79,1.76])^c^ | IRR 1.28  (95% CI [0.85,1.91])^c^ | | IRR 1.24  (95% CI [0.80,1.91])^c^ | | Parental age, education, smoking, income, countries of origin, cohabitation status, place, family history of psychiatric disorders, asthma, parity and preterm delivery, sex, calendar year of follow-up, congenital malformation, childhood history of asthma or asthma treatment |
|  | Non-asthmatic | ASD |  | IRR 1.31  (95% CI [1.13,1.53])^c^ | IRR 1.25  (95% CI [1.01,1.55])^c^ | IRR 1.41  (95% CI [1.14,1.76])^c^ | IRR 1.18  (95% CI [0.91,1.54])^c^ | | Parental age, education, smoking, income, countries of origin, cohabitation status, place, family history of psychiatric disorders, asthma, parity and preterm delivery, sex, calendar year of follow-up, congenital malformation, childhood history of asthma or asthma treatment | |
| Liang H. et al. (2017) | Asthmatic | ADHD |  | IRR 0.86  (95% CI [0.66,1.12])^c^ | IRR 0.49  (95% CI [0.28,0.88])^c^ | IRR 1.00  (95% CI [0.66,1.53])^c^ | IRR 0.92  (95% CI [0.53,1.60])^c^ | | Paternal age at childbirth, maternal age at childbirth, maternal education, maternal smoking during pregnancy, maternal socioeconomic status, parental history of psychiatric disorders, maternal history of asthma before delivery and inhaled glucocorticoid use during pregnancy, birth year, parity and sex | |
|  | Non-asthmatic | ADHD |  | IRR 1.27  (95% CI [1.14,1.42])^c^ | IRR 1.16  (95% CI [0.96,1.40])^c^ | IRR 1.18  (95% CI [0.98,1.42])^c^ | IRR 1.57  (95% CI [1.29,1.90])^c^ | | Paternal age at childbirth, maternal age at childbirth, maternal education, maternal smoking during pregnancy, maternal socioeconomic status, parental history of psychiatric disorders, maternal history of asthma before delivery and inhaled glucocorticoid use during pregnancy, birth year, parity and sex | |
| Nagata et al. (2023) | Asthmatic | Communication problems |  | *Exposure to B2AA:  - Early pregnancy: OR 1.10 (95% CI [0.31,3.86])^c^  - Mid to late pregnancy: OR 1.08 (95% CI [0.50,2.15])^c^  - Both early and mid to late pregnancy: OR 1.46 (95% CI [0.69,3.09])^c^  *Exposure to corticosteroids:  - Early pregnancy: OR 1.44 (95% CI [0.72,2.86])^c^  - Mid to late pregnancy: OR 1.14 (95% CI [0.68,1.91])^c^  - Both early and mid to late pregnancy: OR 1.32 (95% CI [0.79,2.22])^c^ |  |  |  | | Maternal age at delivery, marital status, educational level, alcohol consumption during pregnancy, maternal and paternal smoking during pregnancy, household annual income and offspring | |
|  | Non- asthmatic | Communication problems |  | *Exposure to B2AA:  - Early pregnancy: OR 0.20 (95% CI [0.03,1.45])^c^  - Mid to late pregnancy: OR 1.62 (95% CI [0.83,3.13])^c^  - Both early and mid to late pregnancy: OR 0.49 (95% CI [0.07,3.30])^c^  *Exposure to corticosteroids:  - Early pregnancy: OR 0.63 (95% CI [0.27,1.46])^c^  - Mid to late pregnancy: OR 1.06 (95% CI [0.66,1.68])^c^  - Both early and mid to late pregnancy: OR 1.61 (95% CI [0.86,3.02])^c^ |  |  |  | | Maternal age at delivery, marital status, educational level, alcohol consumption during pregnancy, maternal and paternal smoking during pregnancy, household annual income and offspring | |
|  | Asthmatic | Gross motor |  | *Exposure to B2AA:  - Early pregnancy: OR 1.32 (95% CI [0.67,2.07])^c^  - Mid to late pregnancy: OR 1.11 (95% CI [0.70,1.75])^c^  - Both early and mid to late pregnancy: OR 0.99 (95% CI [0.63,1.55])^c^  *Exposure to corticosteroids:  - Early pregnancy: OR 0.92 (95% CI [0.52,1.61])^c^  - Mid to late pregnancy: OR 1.15 (95% CI [0.84,1.57])^c^  - Both early and mid to late pregnancy: OR 1.17 (95% CI [0.84,1.63])^c^ |  |  |  | | Maternal age at delivery, marital status, educational level, alcohol consumption during pregnancy, maternal and paternal smoking during pregnancy, household annual income and offspring | |
|  | Non-asthmatic | Gross motor |  | *Exposure to B2AA:  - Early pregnancy: OR 0.70 (95% CI [0.25,1.98])^c^  - Mid to late pregnancy: OR 1.46 (95% CI [0.87,2.42])^c^  - Both early and mid to late pregnancy: OR 0.20 (95% CI [0.03,1.35])^c^  *Exposure to corticosteroids:  - Early pregnancy: OR 0.81 (95% CI [0.53,1.22])^c^  - Mid to late pregnancy: OR 0.88 (95% CI [0.64,1.20])^c^  - Both early and mid to late pregnancy: OR 1.14 (95% CI [0.71,1.83])^c^ |  |  |  | | Maternal age at delivery, marital status, educational level, alcohol consumption during pregnancy, maternal and paternal smoking during pregnancy, household annual income and offspring | |
|  | Asthmatic | Fine motor |  | *Exposure to B2AA:  - Early pregnancy: OR 0.65 (95% CI [0.30,3.38])^c^  - Mid to late pregnancy: OR 1.01 (95% CI [0.57,1.79])^c^  - Both early and mid to late pregnancy: OR 0.86 (95% CI [0.52,1.41])^c^  *Exposure to corticosteroids:  - Early pregnancy: OR 0.75 (95% CI [0.45,1.26])^c^  - Mid to late pregnancy: OR 1.03 (95% CI [0.75,1.42])^c^  - Both early and mid to late pregnancy: OR 1.26 (95% CI [0.91,1.71])^c^ |  |  |  | | Maternal age at delivery, marital status, educational level, alcohol consumption during pregnancy, maternal and paternal smoking during pregnancy, household annual income and offspring | |
|  | Non-asthmatic | Fine motor |  | *Exposure to B2AA:  - Early pregnancy: OR 0.84 (95% CI [0.37,1.89])^c^  - Mid to late pregnancy: OR 1.51 (95% CI [0.92,1.42])^c^  - Both early and mid to late pregnancy: OR 0.73 (95% CI [0.17,3.02])^c^  *Exposure to corticosteroids:  - Early pregnancy: OR 1.01 (95% CI [0.68,1.51])^c^  - Mid to late pregnancy: OR 0.83 (95% CI [0.56,1.29])^c^  - Both early and mid to late pregnancy: OR 1.17 (95% CI [0.79,1.75])^c^ |  |  |  | | Maternal age at delivery, marital status, educational level, alcohol consumption during pregnancy, maternal and paternal smoking during pregnancy, household annual income and offspring | |
|  | Asthmatic | Problem solving |  | *Exposure to B2AA:  - Early pregnancy: OR 1.03 (95% CI [0.56,1.91])^c^  - Mid to late pregnancy: OR 1.14 (95% CI [0.71,1.84])^c^  - Both early and mid to late pregnancy: OR 1.09 (95% CI [0.67,1.78])^c^  *Exposure to corticosteroids:  - Early pregnancy: OR 1.06 (95% CI [0.65,1.74])^c^  - Mid to late pregnancy: OR 1.08 (95% CI [0.78,1.50])^c^  - Both early and mid to late pregnancy: OR 1.37 (95% CI [1.01,1.84])^c^ |  |  |  | | Maternal age at delivery, marital status, educational level, alcohol consumption during pregnancy, maternal and paternal smoking during pregnancy, household annual income and offspring | |
|  | Non-asthmatic | Problem solving |  | *Exposure to B2AA:  - Early pregnancy: OR 0.68 (95% CI [0.28,1.65])^c^  - Mid to late pregnancy: OR 1.25 (95% CI [0.77,2.04])^c^  - Both early and mid to late pregnancy: OR 1.01 (95% CI [0.38,2.63])^c^  *Exposure to corticosteroids:  - Early pregnancy: OR 0.93 (95% CI [0.64,1.37])^c^  - Mid to late pregnancy: OR 0.96 (95% CI [0.72,1.27])^c^  - Both early and mid to late pregnancy: OR 0.97 (95% CI [0.62,1.52])^c^ |  |  |  | | Maternal age at delivery, marital status, educational level, alcohol consumption during pregnancy, maternal and paternal smoking during pregnancy, household annual income and offspring | |
|  | Asthmatic | Personal-social |  | *Exposure to B2AA:  - Early pregnancy: OR 0.82 (95% CI [0.20,3.33])^c^  - Mid to late pregnancy: OR 1.56 (95% CI [0.90,2.70])^c^  - Both early and mid to late pregnancy: OR 0.57 (95% CI [0.26,1.25])^c^  *Exposure to corticosteroids:  - Early pregnancy: OR 1.16 (95% CI [0.53,2.52])^c^  - Mid to late pregnancy: OR 1.03 (95% CI [0.66,1.59])^c^  - Both early and mid to late pregnancy: OR 1.11 (95% CI [0.63,1.97])^c^ |  |  |  | | Maternal age at delivery, marital status, educational level, alcohol consumption during pregnancy, maternal and paternal smoking during pregnancy, household annual income and offspring | |
|  | Non-asthmatic | Personal-social |  | *Exposure to B2AA:  - Early pregnancy: OR 0.61 (95% CI [0.14,2.58])^c^  - Mid to late pregnancy: OR 1.47 (95% CI [0.70,3.11])^c^  - Both early and mid to late pregnancy: OR 0.47 (95% CI [0.06,3.24])^c^  *Exposure to corticosteroids:  - Early pregnancy: OR 0.77 (95% CI [0.43,1.39])^c^  - Mid to late pregnancy: OR 0.89 (95% CI [0.54,1.47])^c^  - Both early and mid to late pregnancy: OR 0.66 (95% CI [0.28,1.53])^c^ |  |  |  | | Maternal age at delivery, marital status, educational level, alcohol consumption during pregnancy, maternal and paternal smoking during pregnancy, household annual income and offspring | |

ASD autism spectrum disorder; ADHD attention deficit hyperactivity disorder; BMI body mass index; B2AA beta-2 adrenergic agonists; CI confidence interval; IRR incidence rate ratio; OR odd ratio

^a^ reference group had no exposure during the exposure period of interest

^b^ reference group had asthma but with no medications

^c^ reference group had no exposure during pregnancy

^1^Inhaled B2AA with or without other asthma medications.

# **Table E.** Sub-group analyses of the associations between beta-2-adrenergic agonists and autism spectrum disorder stratified by child’s sex

| Authors and years | Sex | Neurodevelopmental outcomes | Preconception | During pregnancy | 1st Trimester | | 2nd Trimester | 3rd Trimester | Covariates in the adjusted models |
| --- | --- | --- | --- | --- | --- | --- | --- | --- | --- |
| Liang H. et al. (2017) | Boys | ADHD | IRR 1.25  (95% CI [1.15,1.36])^a^ | IRR 1.21  (95% CI [1.08,1.36])^b^ | IRR 1.11  (95% CI [0.90,1.35])^b^ | | IRR 1.11  (95% CI [0.92,1.35])^b^ | IRR 1.57  (95% CI [1.28,1.92])^b^ | Paternal age at childbirth, maternal age at childbirth, maternal education, maternal smoking during pregnancy, maternal socioeconomic status, parental history of psychiatric disorders, maternal history of asthma before delivery and inhaled glucocorticoid use during pregnancy, birth year, parity and sex |
|  | Girls | ADHD | IRR 1.46  (95% CI [1.26,1.69])^a^ | IRR 1.18  (95% CI [0.95,1.46])^b^ | IRR 0.85  (95% CI [0.57,1.28])^b^ | | IRR 1.35  (95% CI [0.98,1.87])^b^ | IRR 1.21  (95% CI [0.80,1.82])^b^ | Paternal age at childbirth, maternal age at childbirth, maternal education, maternal smoking during pregnancy, maternal socioeconomic status, parental history of psychiatric disorders, maternal history of asthma before delivery and inhaled glucocorticoid use during pregnancy, birth year, parity and sex |
| Su X. et al. (2017) | Boys | ASD |  | IRR 1.30  (95% CI [1.11,1.52])^b^ | IRR 1.22  (95% CI [0.98,1.51])^b^ | | IRR 1.36  (95% CI [1.10,1.69])^b^ | IRR 1.31  (95% CI [1.03,1.67])^b^ | Parental age, education, smoking, income, countries of origin, cohabitation status, place, family history of psychiatric disorders, asthma, parity and preterm delivery, sex, calendar year of follow-up, congenital malformation, childhood history of asthma or asthma treatment |
|  | Girls | ASD |  | IRR 1.21  (95% CI [1.04,1.63])^b^ | IRR 1.31  (95% CI [0.87,1.99])^b^ | | IRR 1.46  (95% CI [0.96,2.22])^b^ | IRR 0.73  (95% CI [0.39,1.35])^b^ | Parental age, education, smoking, income, countries of origin, cohabitation status, place, family history of psychiatric disorders, asthma, parity and preterm delivery, sex, calendar year of follow-up, congenital malformation, childhood history of asthma or asthma treatment |
| Li L. et al. (2018) | Boys | Cerebral Palsy | OR 1.04  (95% CI [0.73,1.47])^c^ | OR 0.89  (95% CI [0.56,1.43])^b^ |  | |  |  | Maternal age at delivery, paternal age, maternal education, maternal cohabitation status, maternal smoking status, maternal history of Cerebral Palsy, maternal asthma hospital-diagnosed before delivery, birth year, parity, and sex |
|  | Girls | Cerebral Palsy | OR 1.11  (95% CI [0.74,1.67])^c^ | OR 1.41  (95% CI [0.89,2.22])^b^ |  | |  |  | Maternal age at delivery, paternal age, maternal education, maternal cohabitation status, maternal smoking status, maternal history of Cerebral Palsy, maternal asthma hospital-diagnosed before delivery, birth year, parity, and sex |
| Nagata et al. (2023) | Boys | Communication problems |  | *Exposure to B2AA:  - Early pregnancy: OR 0.11 (95% CI [0.01,0.77])^b^  - Mid to late pregnancy: OR 1.10 (95% CI [1.14,2.16])^b^  - Both early and mid to late pregnancy: OR 0.64 (95% CI [0.16,2.58])^b^  *Exposure to corticosteroids:  - Early pregnancy: OR OR 0.87 (95% CI [0.45,1.69])^b^  - Mid to late pregnancy: OR 1.05 (95% CI [0.70,1.58])^b^  - Both early and mid to late pregnancy: OR 0.99 (95% CI [0.58,1.68])^b^ | |  |  |  | Maternal age at delivery, marital status, educational level, history of pre-pregnancy asthma, alcohol consumption during pregnancy, maternal and paternal smoking during pregnancy, and household annual income. |
|  | Girls | Communication problems |  | *Exposure to B2AA:  - Early pregnancy: OR 2.47 (95% CI [0.70,8.64])^b^  - Mid to late pregnancy: OR 0.79 (95% CI [0.59,1.23])^b^  - Both early and mid to late pregnancy: OR 3.03 (95% CI [1.43,6.45])^b^  *Exposure to Corticosteroids:  - Early pregnancy: OR 1.23 (95% CI [0.50,2.05])^b^  - Mid to late pregnancy: OR 1.08 (95% CI [0.59,1.99])^b^  - Both early and mid to late pregnancy: OR 1.61 (95% CI [0.86,3.02])^b^ |  | |  |  | Maternal age at delivery, marital status, educational level, history of pre-pregnancy asthma, alcohol consumption during pregnancy, maternal and paternal smoking during pregnancy, and household annual income. |
|  | Boys | Gross motor |  | *Exposure to B2AA:  - Early pregnancy: OR 1.33 (95% CI [0.64,2.77])^b^  - Mid to late pregnancy: OR 1.54 (95% CI [0.95,2.50])^b^  - Both early and mid to late pregnancy: OR 0.83 (95% CI [0.47,1.49])^b^  *Exposure to corticosteroids:  - Early pregnancy: OR 0.86 (95% CI [0.53,1.38])^b^  - Mid to late pregnancy: OR 0.98 (95% CI [0.73,1.32])^b^  - Both early and mid to late pregnancy: OR 1.11 (95% CI [0.77,1.59])^b^ |  | |  |  | Maternal age at delivery, marital status, educational level, history of pre-pregnancy asthma, alcohol consumption during pregnancy, maternal and paternal smoking during pregnancy, and household annual income. |
|  | Girls | Gross motor |  | *Exposure to B2AA:  - Early pregnancy: OR 0.71 (95% CI [0.30,1.68])^b^  - Mid to late pregnancy: OR 0.96 (95% CI [0.59,1.57])^b^  - Both early and mid to late pregnancy: OR 0.76 (95% CI [0.40,1.46])^b^  *Exposure to corticosteroids:  - Early pregnancy: OR 0.79 (95% CI [0.48,1.28])^b^  - Mid to late pregnancy: OR 0.93 (95% CI [0.67,1.29])^b^  - Both early and mid to late pregnancy: OR 1.09 (95% CI [0.73,1.64])^b^ |  | |  |  | Maternal age at delivery, marital status, educational level, history of pre-pregnancy asthma, alcohol consumption during pregnancy, maternal and paternal smoking during pregnancy, and household annual income. |
|  | Boys | Fine motor |  | *Exposure to B2AA:  - Early pregnancy: OR 0.51 (95% CI [0.22,1.17])^b^  - Mid to late pregnancy: OR 1.66 (95% CI [1.08,2.54])^b^  - Both early and mid to late pregnancy: OR 0.80 (95% CI [0.45,1.43])^b^  *Exposure to corticosteroids:  - Early pregnancy: OR 1.02 (95% CI [0.71,1.45])^b^  - Mid to late pregnancy: OR 0.99 (95% CI [0.75,1.29])^b^  - Both early and mid to late pregnancy: OR 1.19 (95% CI [0.87,1.62])^b^ |  | |  |  | Maternal age at delivery, marital status, educational level, history of pre-pregnancy asthma, alcohol consumption during pregnancy, maternal and paternal smoking during pregnancy, and household annual income. |
|  | Girls | Fine motor |  | *Exposure to B2AA:  - Early pregnancy: OR 1.00 (95% CI [0.47,2.13])^b^  - Mid to late pregnancy: OR 0.63 (95% CI [0.30,1.30])^b^  - Both early and mid to late pregnancy: OR 0.75 (95% CI [0.33,1.69])^b^  *Exposure to corticosteroids:  - Early pregnancy: OR 0.56 (95% CI [0.28,1.11])^b^  - Mid to late pregnancy: OR 0.83 (95% CI [0.56,1.29])^b^  - Both early and mid to late pregnancy: OR 1.12 (95% CI [0.74,1.68])^b^ |  | |  |  | Maternal age at delivery, marital status, educational level, history of pre-pregnancy asthma, alcohol consumption during pregnancy, maternal and paternal smoking during pregnancy, and household annual income. |
|  | Boys | Problem solving |  | *Exposure to B2AA:  - Early pregnancy: OR 0.66 (95% CI [0.34,1.28])^b^  - Mid to late pregnancy: OR 1.65 (95% CI [1.11,2.45])^b^  - Both early and mid to late pregnancy: OR 0.84 (95% CI [0.47,1.51])^b^  *Exposure to corticosteroids:  - Early pregnancy: OR 1.08 (95% CI [0.75,1.56])^b^  - Mid to late pregnancy: OR 0.99 (95% CI [0.77,1.29])^b^  - Both early and mid to late pregnancy: OR 1.11 (95% CI [0.81,1.52])^b^ |  | |  |  | Maternal age at delivery, marital status, educational level, history of pre-pregnancy asthma, alcohol consumption during pregnancy, maternal and paternal smoking during pregnancy, and household annual income. |
|  | Girls | Problem solving |  | *Exposure to B2AA:  - Early pregnancy: OR 1.12 (95% CI [0.52,2.02])^b^  - Mid to late pregnancy: OR 0.55 (95% CI [0.30,0.99])^b^  - Both early and mid to late pregnancy: OR 1.25 (95% CI [0.52,2.40])^b^  *Exposure to corticosteroids:  - Early pregnancy: OR 0.71 (95% CI [0.41,1.22])^b^  - Mid to late pregnancy: OR 0.90 (95% CI [0.62,1.29])^b^  - Both early and mid to late pregnancy: OR 1.16 (95% CI [0.79,1.70])^b^ |  | |  |  | Maternal age at delivery, marital status, educational level, history of pre-pregnancy asthma, alcohol consumption during pregnancy, maternal and paternal smoking during pregnancy, and household annual income. |
|  | Boys | Personal social |  | *Exposure to B2AA:  - Early pregnancy: OR 0.45 (95% CI [0.14,1.46])^b^  - Mid to late pregnancy: OR 1.70 (95% CI [0.97,2.96])^b^  - Both early and mid to late pregnancy: OR 0.42 (95% CI [0.16,1.10])^b^  *Exposure to corticosteroids:  - Early pregnancy: OR 0.99 (95% CI [0.55,1.78])^b^  - Mid to late pregnancy: OR 0.83 (95% CI [0.56,1.22])^b^  - Both early and mid to late pregnancy: OR 0.87 (95% CI [0.51,1.51])^b^ |  | |  |  | Maternal age at delivery, marital status, educational level, history of pre-pregnancy asthma, alcohol consumption during pregnancy, maternal and paternal smoking during pregnancy, and household annual income. |
|  | Girls | Personal social |  | *Exposure to B2AA:  - Early pregnancy: OR 1.35 (95% CI [0.26,6.92])^b^  - Mid to late pregnancy: OR 1.09 (95% CI [0.53,2.23])^b^  - Both early and mid to late pregnancy: OR 0.78 (95% CI [0.25,2.36])^b^  *Exposure to corticosteroids:  - Early pregnancy: OR 0.70 (95% CI [0.30,1.61])^b^  - Mid to late pregnancy: OR 1.12 (95% CI [0.59,2.15])^b^  - Both early and mid to late pregnancy: OR 0.96 (95% CI [0.39,2.33])^b^ |  | |  |  | Maternal age at delivery, marital status, educational level, history of pre-pregnancy asthma, alcohol consumption during pregnancy, maternal and paternal smoking during pregnancy, and household annual income. |

ASD autism spectrum disorder; ADHD attention deficit hyperactivity disorder; B2AA beta-2 adrenergic agonists; CI confidence interval; IRR incidence rate ratio; OR odd ratio

^a^ reference group had no exposure to B2AA from 2 years before pregnancy through delivery

^b^ reference group had no exposure during pregnancy

^c^ reference group had never used B2AA

# **Table F.** Test of heterogeneity and publication bias

| Stage of exposure | Cochran’s Q | P-value of Cochran’s Q | Value of I^2^ | 95% CI of I^2^ | P-value of Egger’s test |
| --- | --- | --- | --- | --- | --- |
| Preconception | 0.51 | 0.774 | 0% | 0% – 57.4% | 0.602 |
| During pregnancy | 0.09 | 0.956 | 0% | 0% - 0% | 0.805 |
| 1^st^ trimester | 0.97 | 0.616 | 0% | 0% - 57.5% | 0.600 |
| 2^nd^ trimester | 0.32 | 0.852 | 0% | 0% - 45.1% | 0.985 |
| 3^rd^ trimester | 1.24 | 0.539 | 0% | 0% - 59% | 0.566 |

# **Table G.** Test of heterogeneity and publication bias for the fixed-effects model

| Stage of exposure | Cochran’s Q | P-value of Cochran’s Q | Value of I^2^ | 95% CI of I^2*^ | P-value of Egger’s test |
| --- | --- | --- | --- | --- | --- |
| Preconception | 0.51 | 0.774 | 0% | 0%-72.9% | 0.602 |
| During pregnancy | 0.09 | 0.956 | 0% | 0%-72.9% | 0.805 |
| 1^st^ trimester | 0.97 | 0.616 | 0% | 0%-72.9% | 0.600 |
| 2^nd^ trimester | 0.32 | 0.852 | 0% | 0%-72.9% | 0.985 |
| 3^rd^ trimester | 1.24 | 0.539 | 0% | 0%-72.9% | 0.566 |

*Same I^2^ confidence intervals across different timings of exposure are due to the limited number of included studies (N=3), in addition to the nature of fixed-effect model that does not estimate between-study variance.

# **Fig A.** Forest plots for each random effects meta-analysis by trimester of pregnancy after excluding study by Croen et al.*

***Preconception***


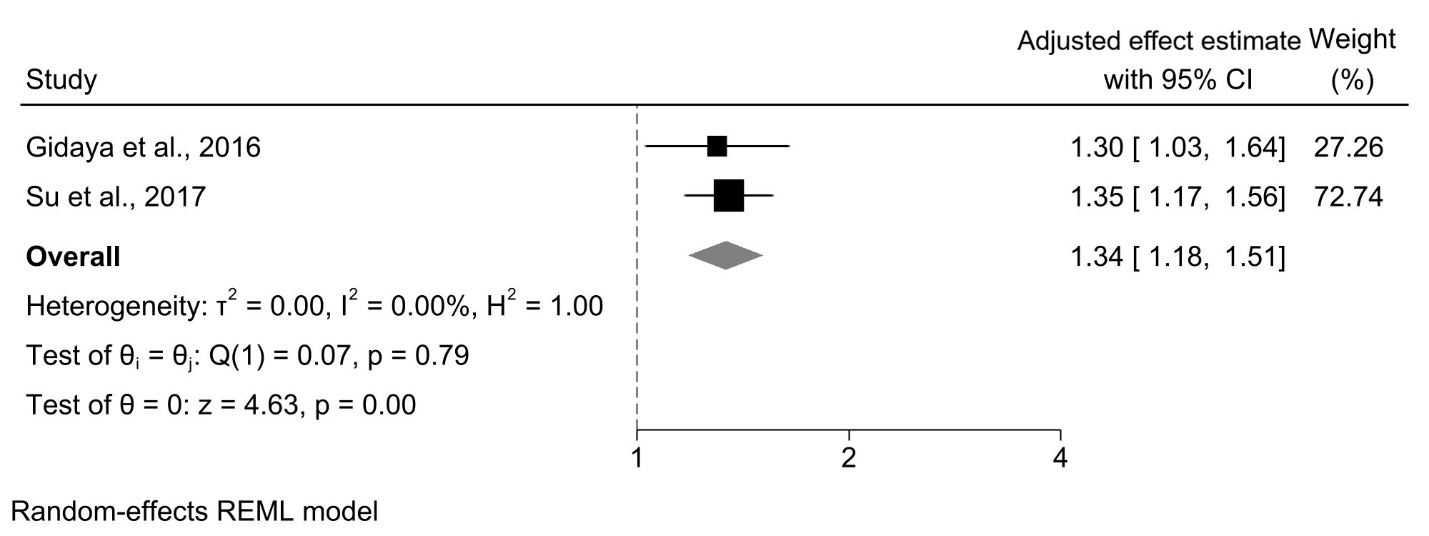


***During pregnancy***


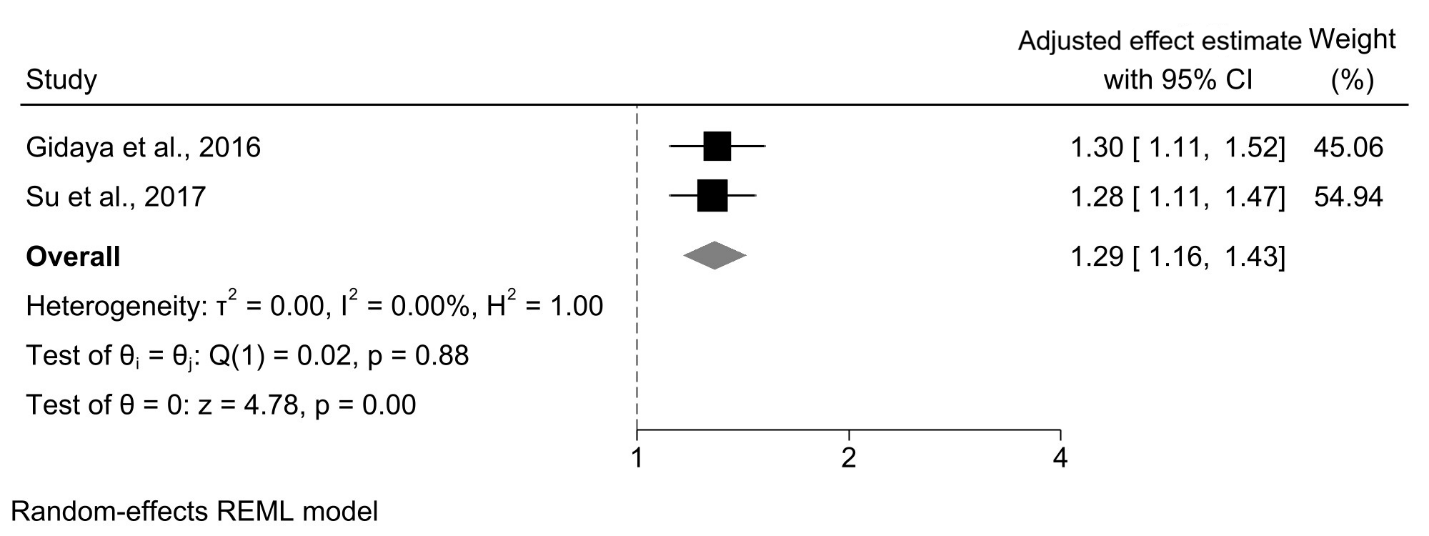


***1st trimester***


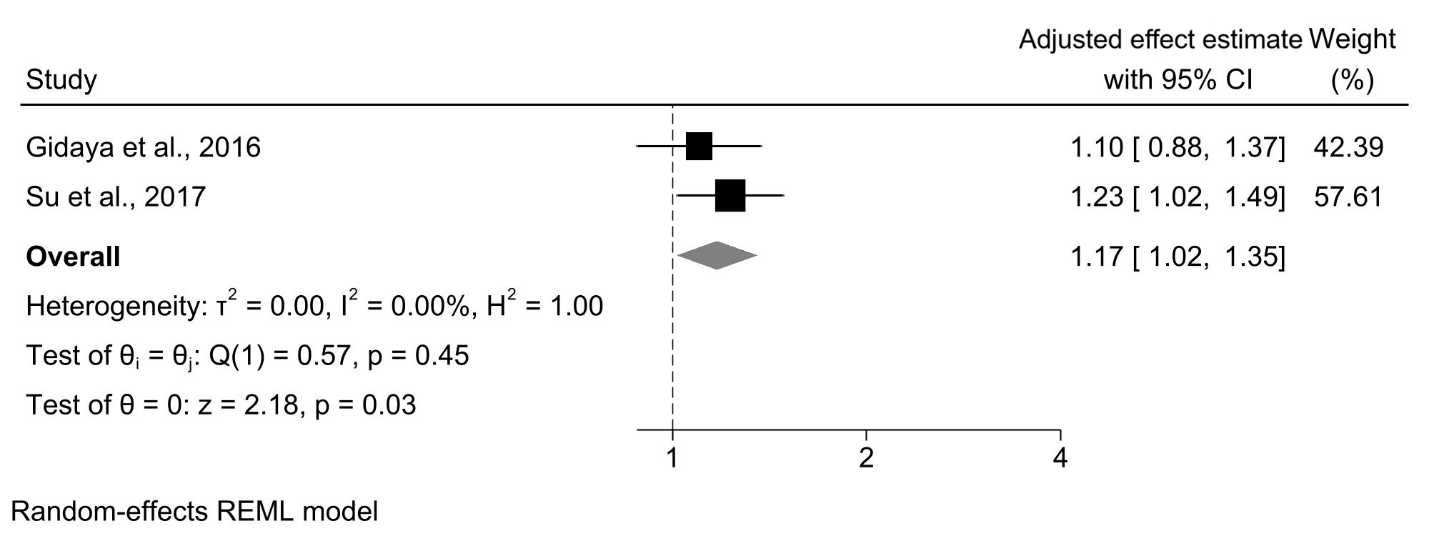


***2nd trimester***


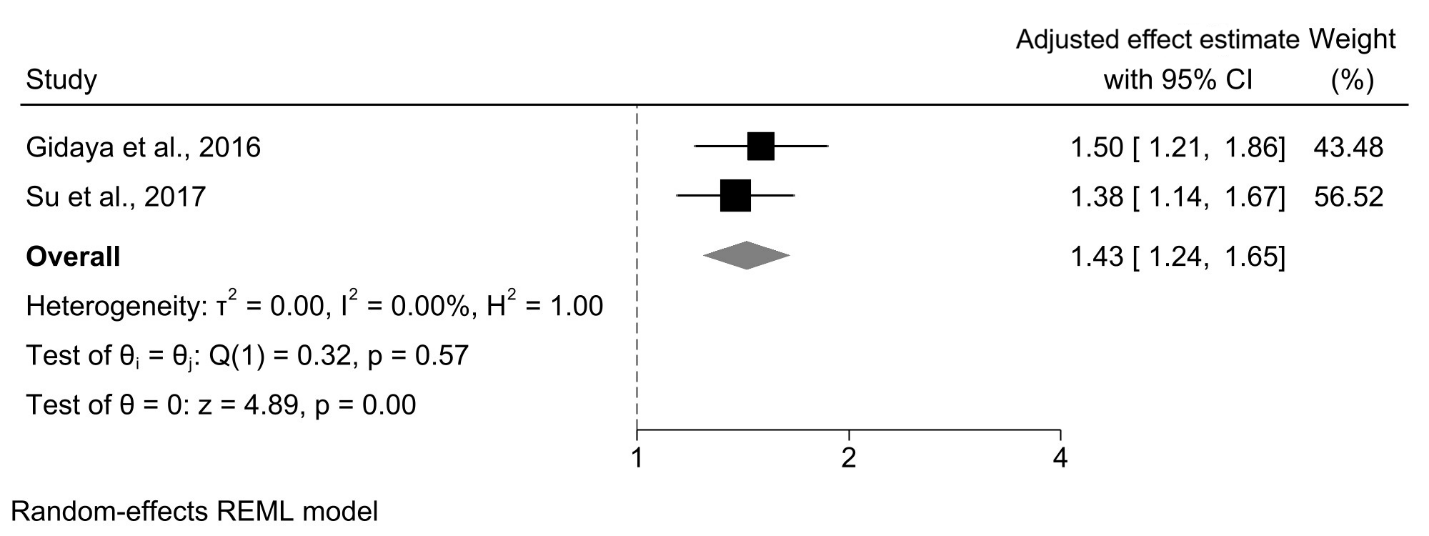


***3rd trimester***

**
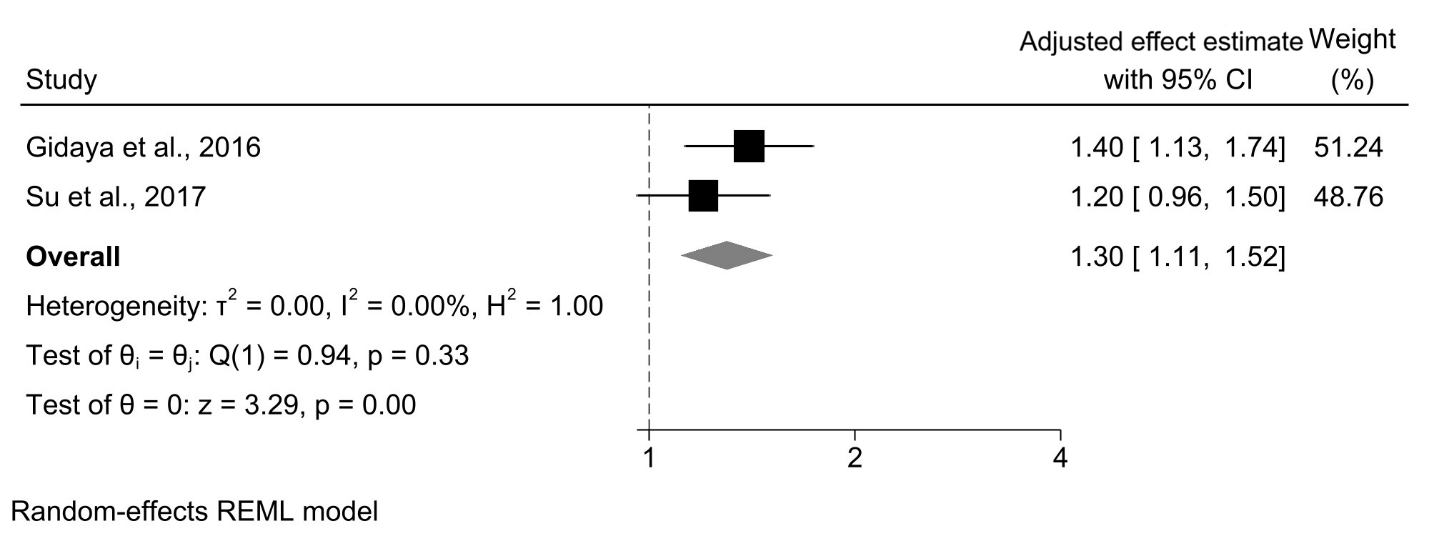
**

*Effect estimates are adjusted measures reported by each study: IRR incidence rate ratio; OR odds ratio. Gidaya et al. reported ORs and Su et al. reported IRRs.

# **Fig B.** Funnel plots for each random effects meta-analysis by trimester of pregnancy after excluding study by Croen et al.

***Preconception***

**
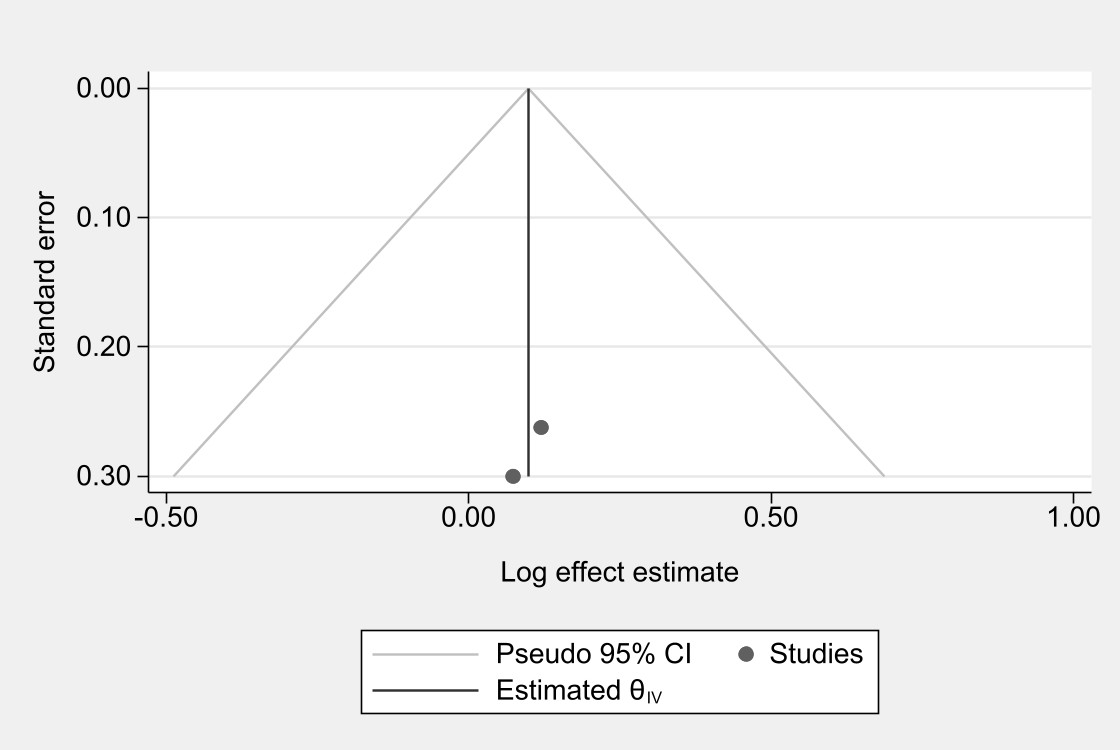
**

***During pregnancy***


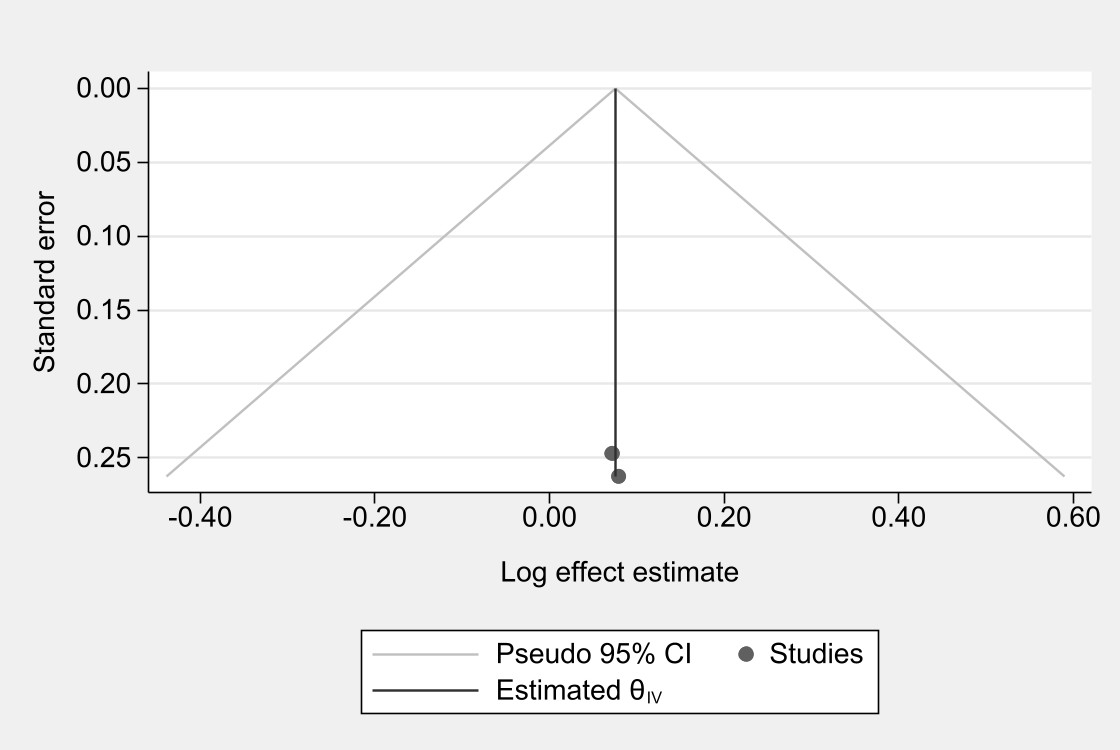


***1^st^ trimester***


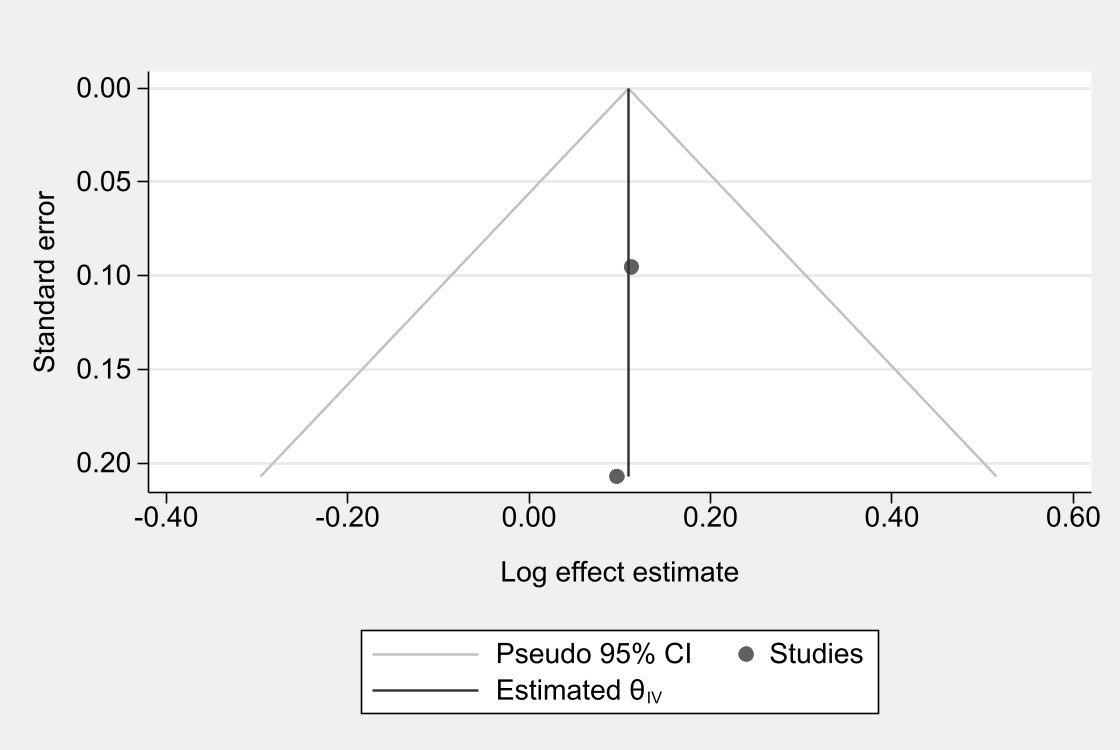


***2^nd^ trimester***

***
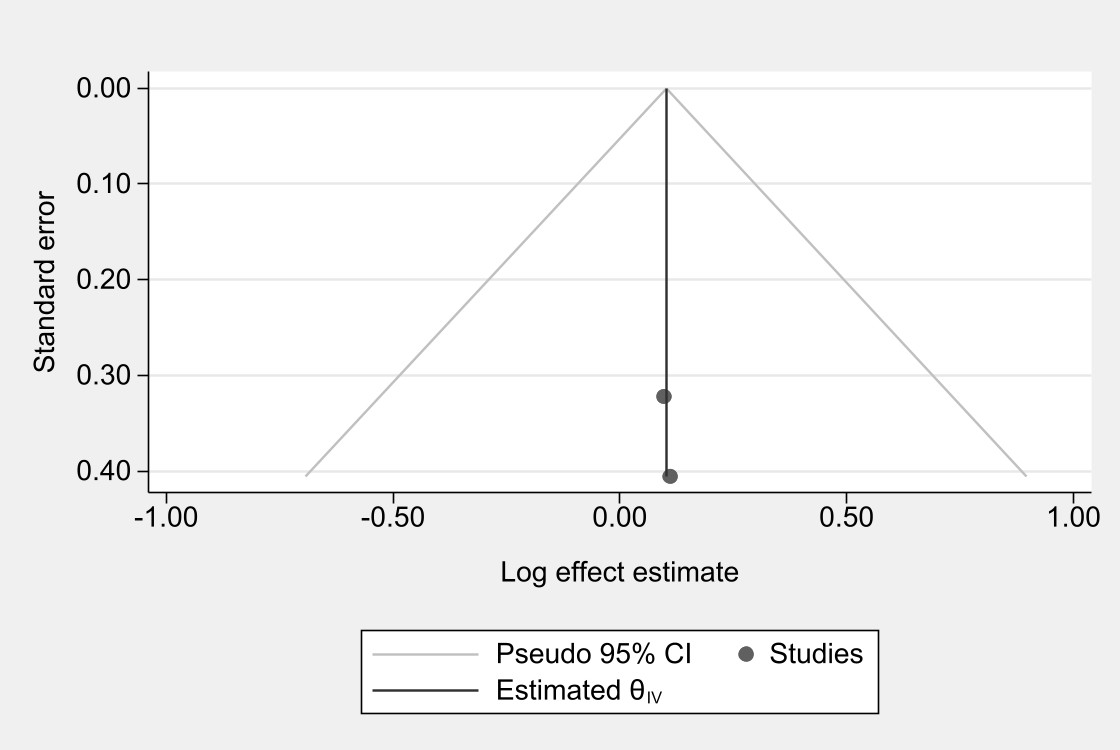
***

***3^rd^ trimester***


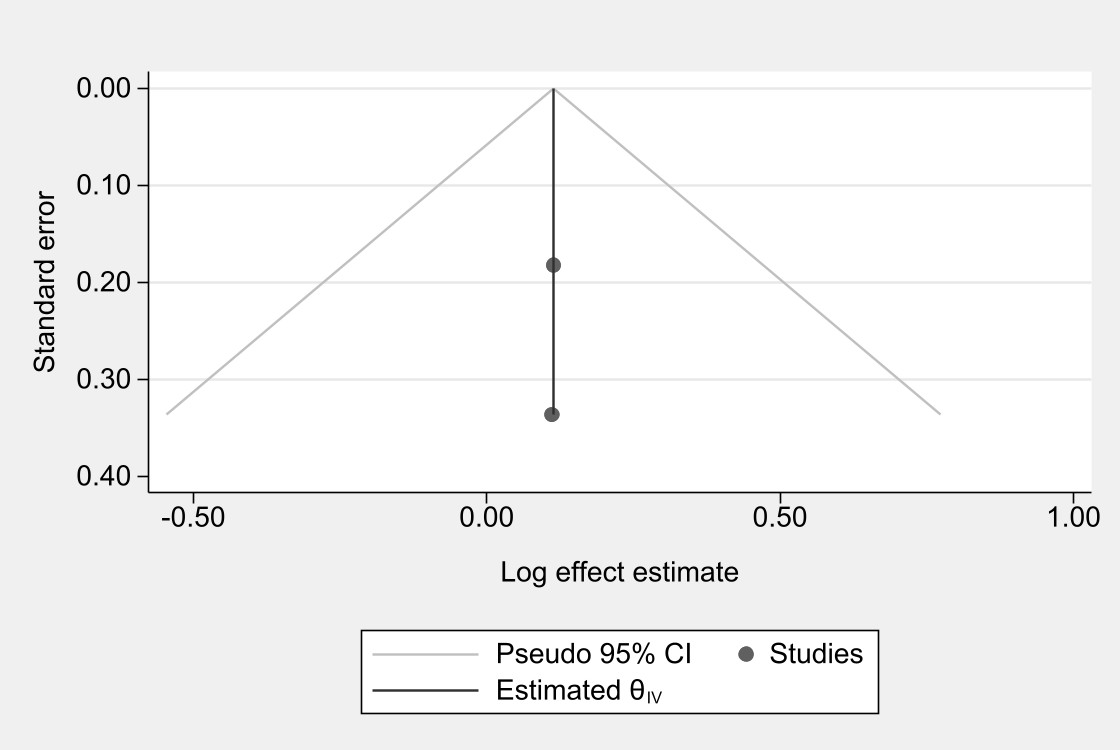


# **Fig C.** Forest plots for each fixed effects meta-analysis by trimester of pregnancy*

***Preconception***


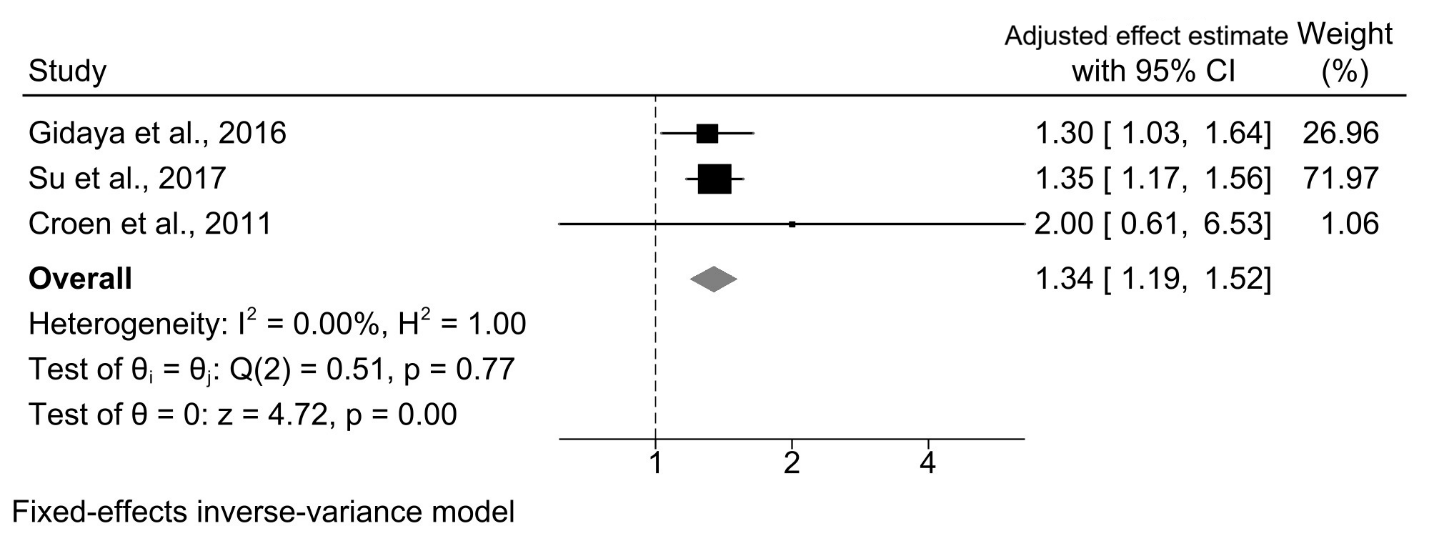


***During pregnancy***


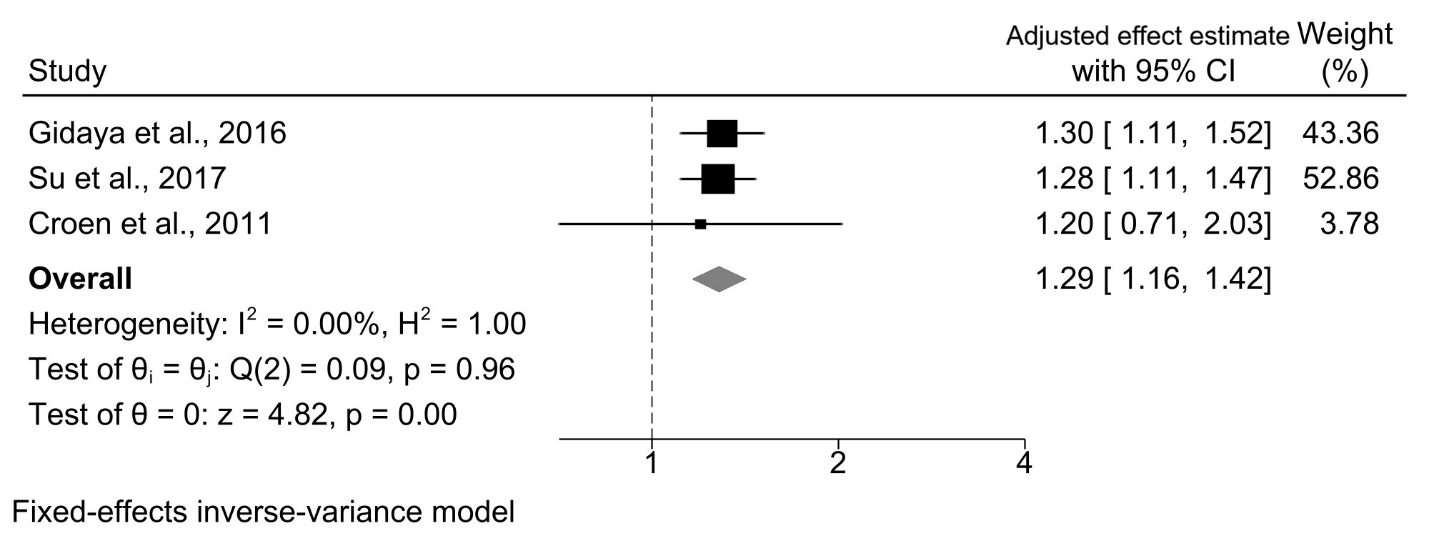


***1st trimester***


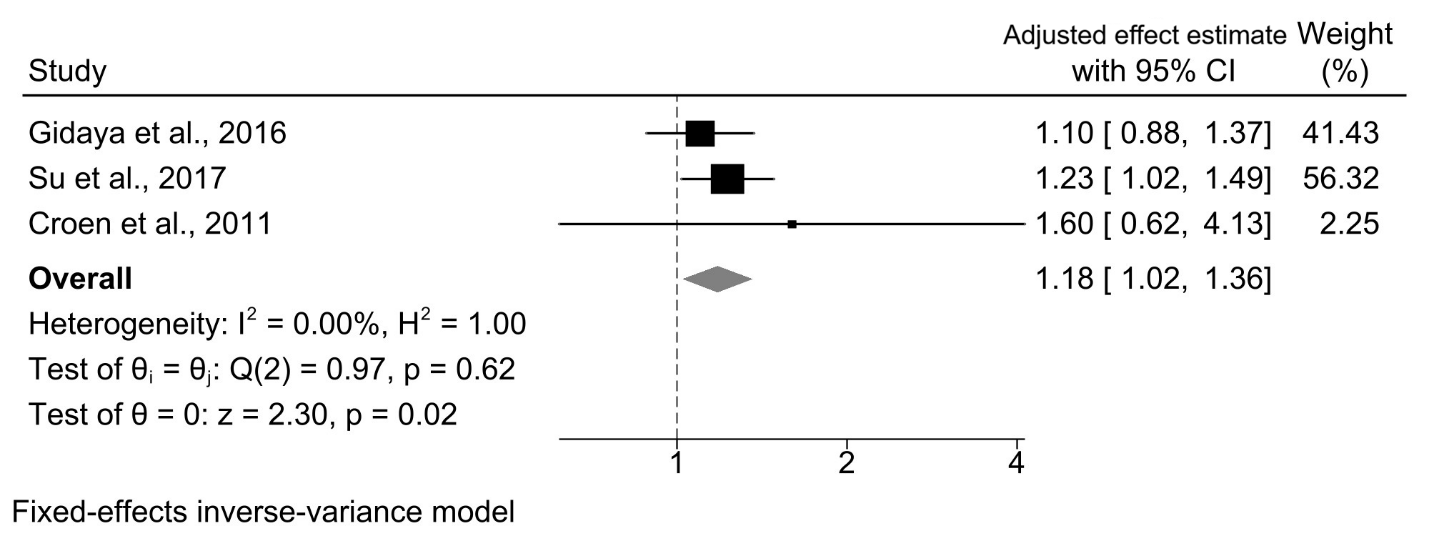


***2nd trimester***


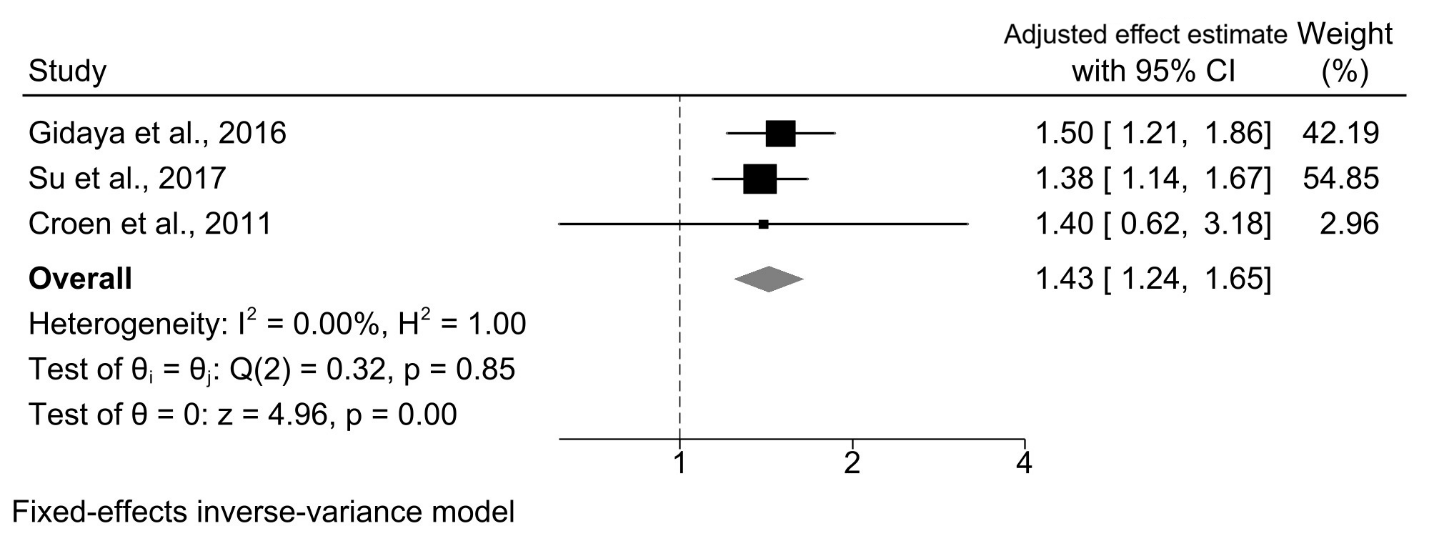


***3rd trimester***


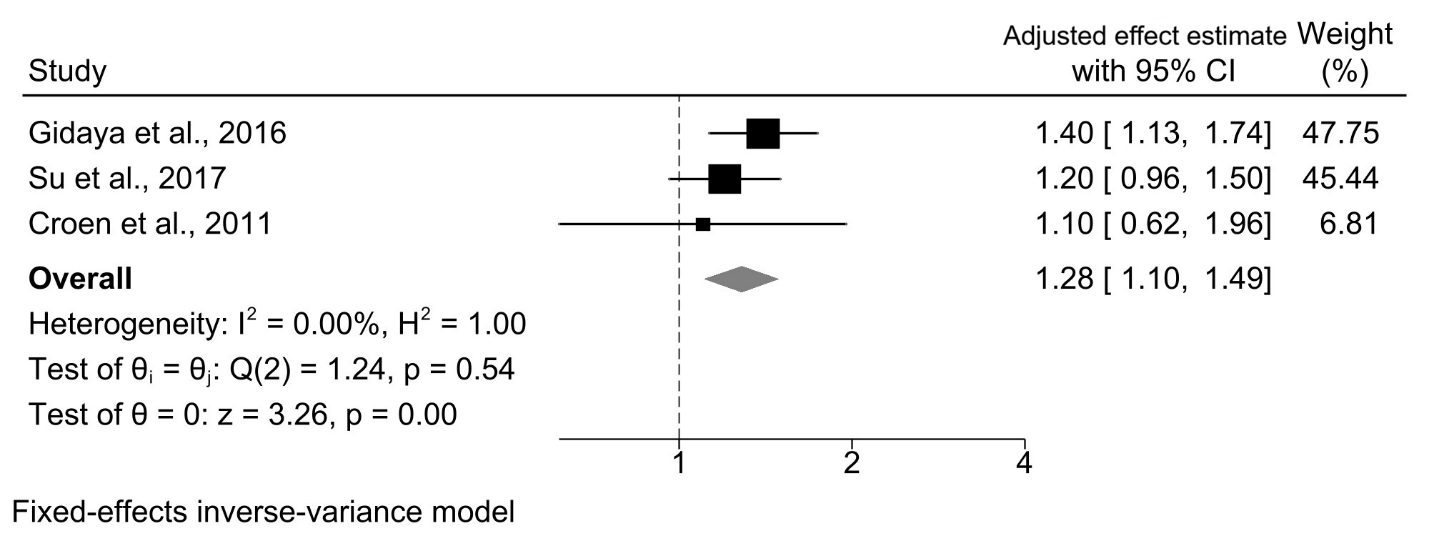


*Effect estimates are adjusted measures reported by each study: IRR incidence rate ratio; OR odds ratio. Su et al. reported IRRs, whereas Gidaya et al. and Croen et al. reported ORs.

# **Fig D.** Funnel plots for each fixed effects meta-analysis by trimester of pregnancy

***Preconception***


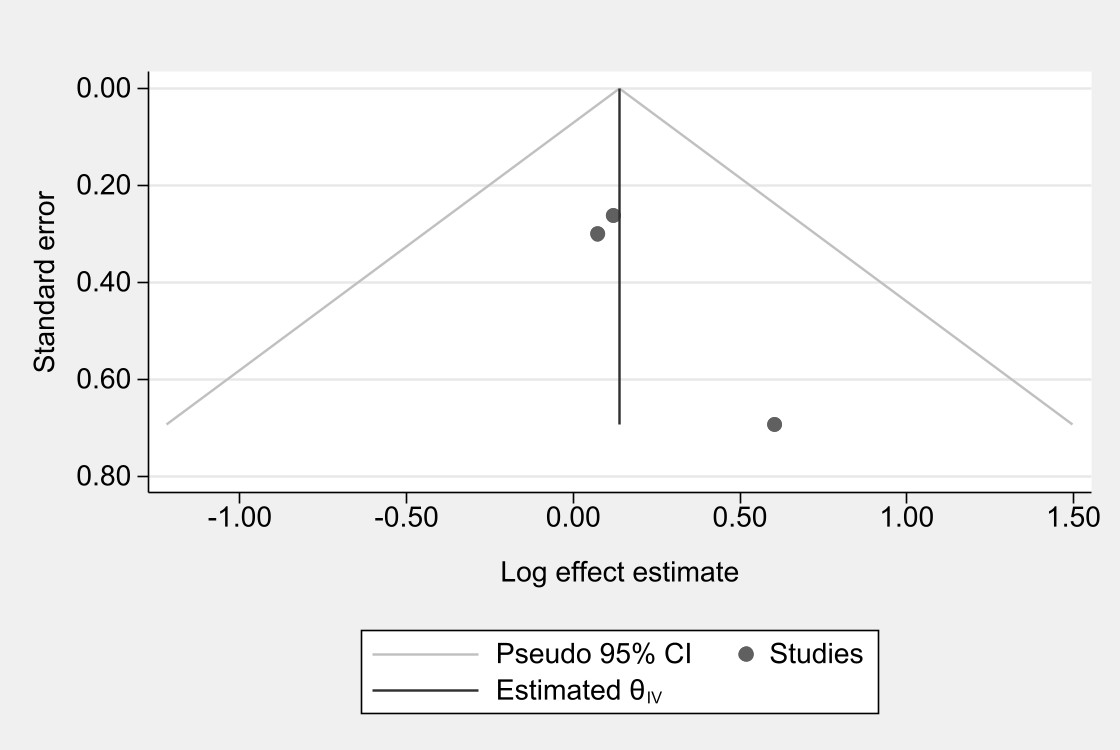


***During pregnancy***


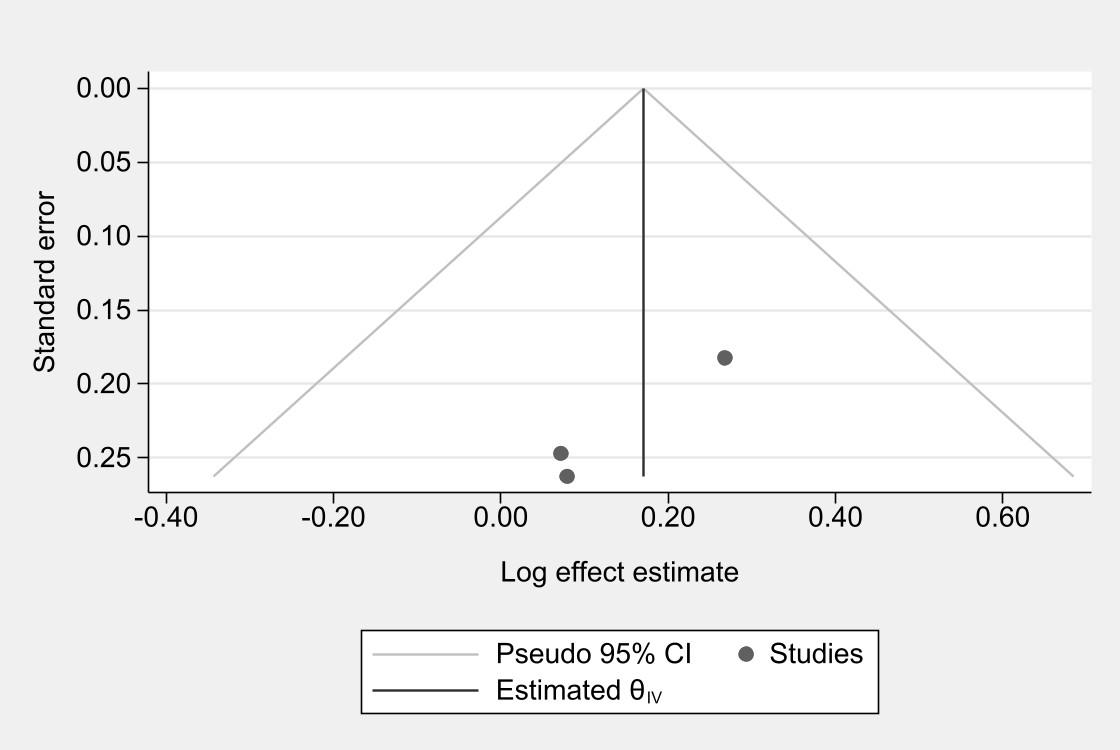


***1st trimester***


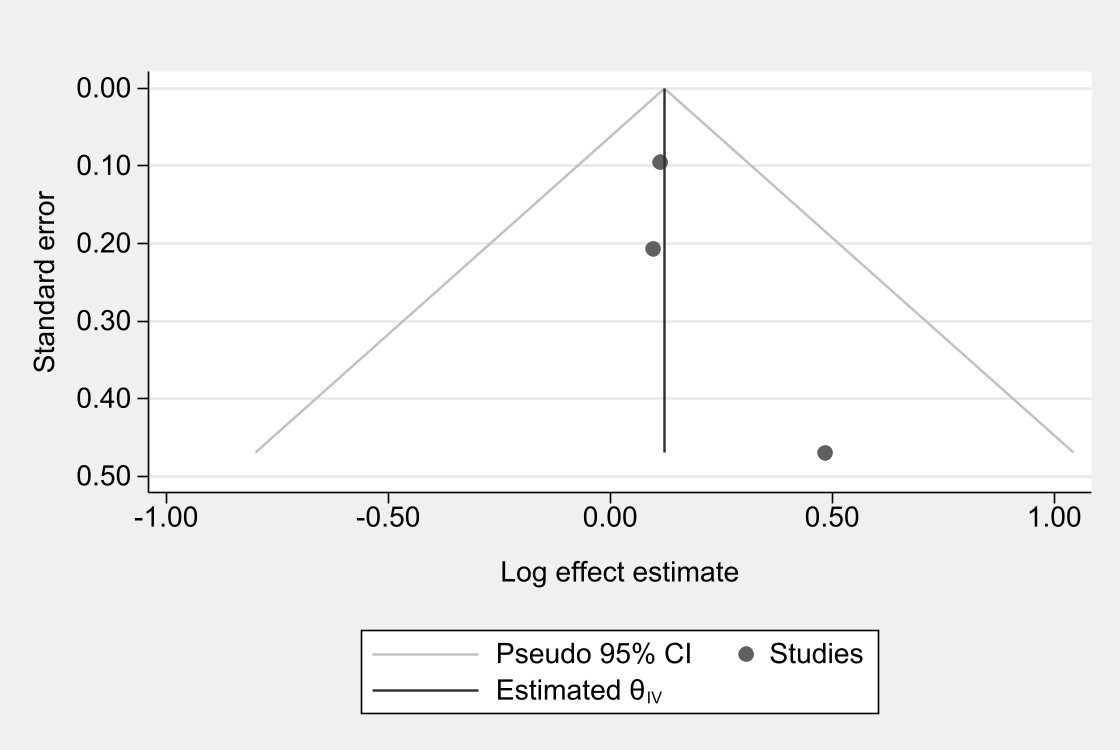


***2nd trimester***


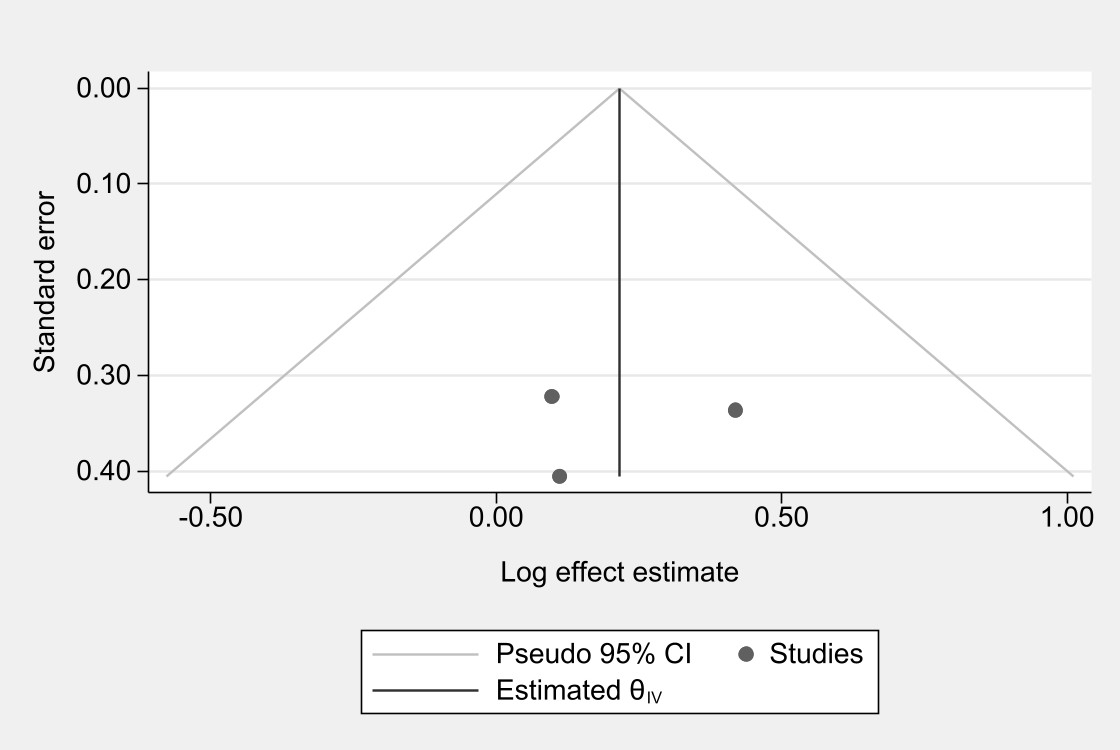


***3rd trimester***


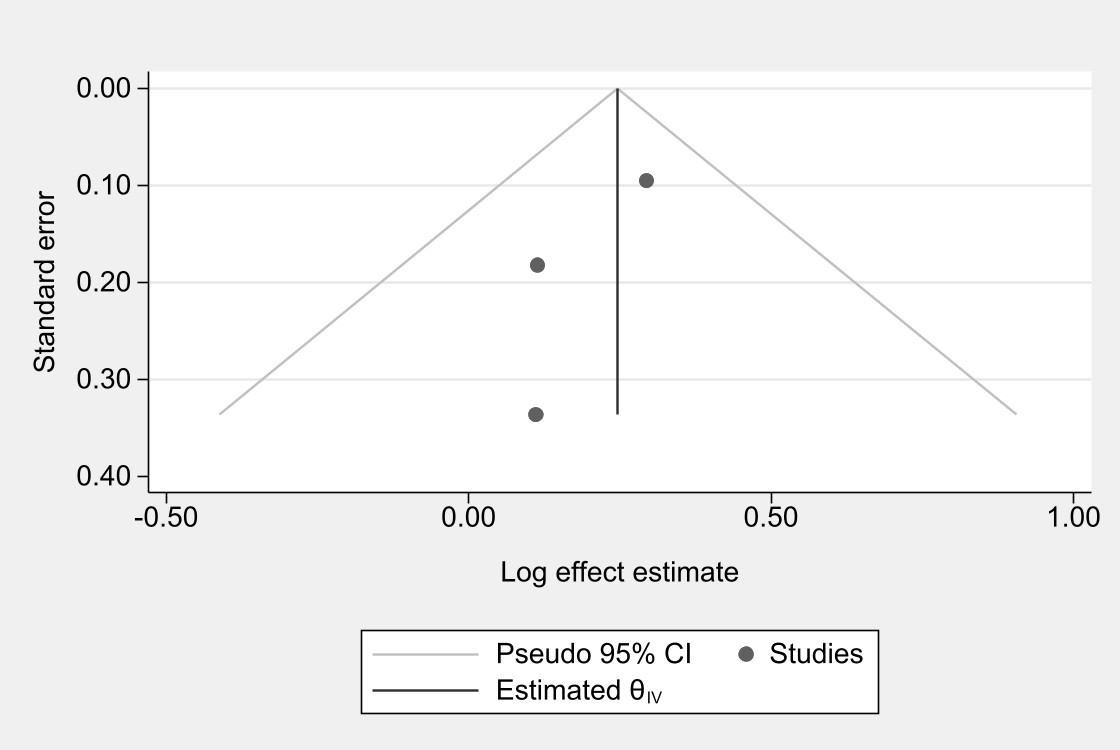


# **Checklist A.** PRISMA 2020 Checklist

| **Section and Topic** | **Item #** | **Checklist item** | **Location where item is reported** |
| --- | --- | --- | --- |
| **TITLE** | | |  |
| Title | 1 | Identify the report as a systematic review. | Title of the manuscript |
| **ABSTRACT** | | |  |
| Abstract | 2 | See the PRISMA 2020 for Abstracts checklist. | Abstract of the manuscript |
| **INTRODUCTION** | | |  |
| Rationale | 3 | Describe the rationale for the review in the context of existing knowledge. | Introduction section, paragraphs 3-6. |
| Objectives | 4 | Provide an explicit statement of the objective(s) or question(s) the review addresses. | Introduction section, paragraph 7. |
| **METHODS** | | |  |
| Eligibility criteria | 5 | Specify the inclusion and exclusion criteria for the review and how studies were grouped for the syntheses. | Methods section, paragraph 2, 5 and 6. |
| Information sources | 6 | Specify all databases, registers, websites, organisations, reference lists and other sources searched or consulted to identify studies. Specify the date when each source was last searched or consulted. | Methods section, paragraphs 1 and 2. |
| Search strategy | 7 | Present the full search strategies for all databases, registers and websites, including any filters and limits used. | S1 Table in the Supporting information file. |
| Selection process | 8 | Specify the methods used to decide whether a study met the inclusion criteria of the review, including how many reviewers screened each record and each report retrieved, whether they worked independently, and if applicable, details of automation tools used in the process. | Methods section, paragraphs 2 and 3. |
| Data collection process | 9 | Specify the methods used to collect data from reports, including how many reviewers collected data from each report, whether they worked independently, any processes for obtaining or confirming data from study investigators, and if applicable, details of automation tools used in the process. | Methods section, paragraph 4. |
| Data items | 10a | List and define all outcomes for which data were sought. Specify whether all results that were compatible with each outcome domain in each study were sought (e.g. for all measures, time points, analyses), and if not, the methods used to decide which results to collect. | Methods section, paragraph 4. |
|  | 10b | List and define all other variables for which data were sought (e.g. participant and intervention characteristics, funding sources). Describe any assumptions made about any missing or unclear information. | Methods section, paragraph 4. |
| Study risk of bias assessment | 11 | Specify the methods used to assess risk of bias in the included studies, including details of the tool(s) used, how many reviewers assessed each study and whether they worked independently, and if applicable, details of automation tools used in the process. | Methods section, paragraphs 7 and 8. |
| Effect measures | 12 | Specify for each outcome the effect measure(s) (e.g. risk ratio, mean difference) used in the synthesis or presentation of results. | Methods section, paragraph 5. |
| Synthesis methods | 13a | Describe the processes used to decide which studies were eligible for each synthesis (e.g. tabulating the study intervention characteristics and comparing against the planned groups for each synthesis (item #5)). | Methods section, paragraph 5. |
|  | 13b | Describe any methods required to prepare the data for presentation or synthesis, such as handling of missing summary statistics, or data conversions. | Methods section, paragraph 5. |
|  | 13c | Describe any methods used to tabulate or visually display results of individual studies and syntheses. | Methods section, paragraph 5. |
|  | 13d | Describe any methods used to synthesize results and provide a rationale for the choice(s). If meta-analysis was performed, describe the model(s), method(s) to identify the presence and extent of statistical heterogeneity, and software package(s) used. | Methods section, paragraphs 5 and 6. |
|  | 13e | Describe any methods used to explore possible causes of heterogeneity among study results (e.g. subgroup analysis, meta-regression). | NA |
|  | 13f | Describe any sensitivity analyses conducted to assess robustness of the synthesized results. | Methods section, paragraph 6. |
| Reporting bias assessment | 14 | Describe any methods used to assess risk of bias due to missing results in a synthesis (arising from reporting biases). | NA |
| Certainty assessment | 15 | Describe any methods used to assess certainty (or confidence) in the body of evidence for an outcome. | Methods section, paragraph 6. |
| **RESULTS** | | |  |
| Study selection | 16a | Describe the results of the search and selection process, from the number of records identified in the search to the number of studies included in the review, ideally using a flow diagram. | Figure 1, and Results section, paragraph 1. |
|  | 16b | Cite studies that might appear to meet the inclusion criteria, but which were excluded, and explain why they were excluded. | Figure 1. |
| Study characteristics | 17 | Cite each included study and present its characteristics. | Table 1, and Results section, paragraph 2. |
| Risk of bias in studies | 18 | Present assessments of risk of bias for each included study. | S2 Table, and Results section, paragraph 2. |
| Results of individual studies | 19 | For all outcomes, present, for each study: (a) summary statistics for each group (where appropriate) and (b) an effect estimate and its precision (e.g. confidence/credible interval), ideally using structured tables or plots. | Table 2, and Results section, paragraphs 5,6 and 8. |
| Results of syntheses | 20a | For each synthesis, briefly summarise the characteristics and risk of bias among contributing studies. | Results section, paragraph 9. |
|  | 20b | Present results of all statistical syntheses conducted. If meta-analysis was done, present for each the summary estimate and its precision (e.g. confidence/credible interval) and measures of statistical heterogeneity. If comparing groups, describe the direction of the effect. | Figure 2, and in the Results section, paragraphs 9 and 10. |
|  | 20c | Present results of all investigations of possible causes of heterogeneity among study results. | NA |
|  | 20d | Present results of all sensitivity analyses conducted to assess the robustness of the synthesized results. | Results section, paragraph 11 |
| Reporting biases | 21 | Present assessments of risk of bias due to missing results (arising from reporting biases) for each synthesis assessed. | NA |
| Certainty of evidence | 22 | Present assessments of certainty (or confidence) in the body of evidence for each outcome assessed. | NA |
| **DISCUSSION** | | |  |
| Discussion | 23a | Provide a general interpretation of the results in the context of other evidence. | Discussion section, paragraphs 1 and 2. |
|  | 23b | Discuss any limitations of the evidence included in the review. | Discussion section, paragraphs 3 - 5. |
|  | 23c | Discuss any limitations of the review processes used. | Discussion section, paragraphs 1, 5 and 7. |
|  | 23d | Discuss implications of the results for practice, policy, and future research. | Discussion section, paragraph 6, and Conclusion section. |
| **OTHER INFORMATION** | | |  |
| Registration and protocol | 24a | Provide registration information for the review, including register name and registration number, or state that the review was not registered. | Methods section, paragraph 2. |
|  | 24b | Indicate where the review protocol can be accessed, or state that a protocol was not prepared. | Methods section, paragraph 2. |
|  | 24c | Describe and explain any amendments to information provided at registration or in the protocol. | NA |
| Support | 25 | Describe sources of financial or non-financial support for the review, and the role of the funders or sponsors in the review. | Funding section. |
| Competing interests | 26 | Declare any competing interests of review authors. | Competing interests section. |
| Availability of data, code and other materials | 27 | Report which of the following are publicly available and where they can be found: template data collection forms; data extracted from included studies; data used for all analyses; analytic code; any other materials used in the review. | Availability of data and materials section. |

*PRISMA 2020 Checklist. Reproduced under CC BY 4.0 from* [*https://www.prisma-statement.org/*](https://www.prisma-statement.org/) *and* Page MJ, McKenzie JE, Bossuyt PM, Boutron I, Hoffmann TC, Mulrow CD, et al. The PRISMA 2020 statement: an updated guideline for reporting systematic reviews. BMJ 2021;372:n71. doi:[10.1136/bmj.n71](https://doi.org/10.1136/bmj.n71)
